# Longitudinal self-concept development in adolescence

**DOI:** 10.1093/scan/nsac062

**Published:** 2023-01-14

**Authors:** Renske van der Cruijsen, Neeltje E Blankenstein, Jochem P Spaans, Sabine Peters, Eveline A Crone

**Affiliations:** Erasmus School of Social and Behavioural Sciences, Erasmus University Rotterdam, Burgemeester Oudlaan 50, Rotterdam 3062 PA, The Netherlands; Department of Developmental Psychology, Leiden University, Wassenaarseweg 52, Leiden 2333 AK, The Netherlands; Leiden Institute for Brain and Cognition, Wassenaarseweg 52, Leiden 2333 AK, The Netherlands; Erasmus School of Social and Behavioural Sciences, Erasmus University Rotterdam, Burgemeester Oudlaan 50, Rotterdam 3062 PA, The Netherlands; Department of Developmental Psychology, Leiden University, Wassenaarseweg 52, Leiden 2333 AK, The Netherlands; Leiden Institute for Brain and Cognition, Wassenaarseweg 52, Leiden 2333 AK, The Netherlands; Erasmus School of Social and Behavioural Sciences, Erasmus University Rotterdam, Burgemeester Oudlaan 50, Rotterdam 3062 PA, The Netherlands; Department of Developmental Psychology, Leiden University, Wassenaarseweg 52, Leiden 2333 AK, The Netherlands

**Keywords:** self-concept, adolescence, longitudinal, fMRI, medial prefrontal cortex, TPJ

## Abstract

This longitudinal behavioral neuroimaging study tested two hypotheses concerning self-concept development in adolescence: domain-specific self-concept and similarity between own (direct) and perceived peers’ (reflected) opinions of the self. Participants (*N* = 189; 10–24 years) evaluated their traits in academic, physical appearance and prosocial domains from direct and reflected perspectives in an functional magnetic resonance imaging session across three time points (TP1: *n* = 160; TP2: *n* = 151; TP3: *n* = 144). Behaviorally, we observed a mid-adolescent dip in self-concept positivity, which was strongest for the academic domain, showing domain differentiation in mid-adolescence. Self-evaluations were associated with activity in, e.g. medial prefrontal cortex (mPFC) and temporal–parietal junction (TPJ). mPFC showed an adolescent-emerging peak in activation, pronounced more for direct than reflected self-evaluations. TPJ activation was generally stronger for reflected self-evaluations, and activation linearly increased with age for both reflected and direct self-evaluations. Longitudinal prediction analyses showed that positivity of self-evaluations predicted increases in self-concept clarity and less fear of negative evaluation 1 and 2 years later, highlighting the developmental benefits of acquiring a positive self-concept. Together, we show that adolescent self-development is characterized by dissociable neural patterns underlying self-evaluations in different domains, and from reflected and direct perspectives, confirming adolescence as a formative phase for developing a coherent and positive self-concept.

## Introduction

Adolescence is the transitional phase between childhood and adulthood and encompasses approximately the age period of 10 to 24 years (Sawyer *et al.*, 2018). A central task during this period is to shape the concept of self in order to make life decisions, such as study or career choices (van der Aar *et al.*, 2019), and to maintain motivation, performance and well-being (Valentine *et al.*, 2004; Huang, 2011; Wouters *et al.*, 2011). Self-concept—the knowledge and beliefs one has about the self (Sebastian *et al.*, 2008; Harter, 2012)—can be subdivided into self-concept clarity (SCC) (the extent to which self-concept is clearly defined, internally consistent and temporally stable) and self-appraisal, which is the estimation or evaluation of one’s positive and negative characteristics (Pfeifer and Peake, 2012; Schwartz *et al.*, 2012; Crone *et al.*, 2022). Self-appraisals can be positive or negative to a smaller or larger extent. Prior functional magnetic resonance imaging (fMRI) studies have combined traditional self-reported evaluations of one’s traits with neuroimaging methods, measuring activation of brain regions while processing self-related stimuli. The combined use of these methods can clarify possible processes associated with the emergence of a stable self-concept. In the current three-wave accelerated longitudinal fMRI study, we assessed adolescent-specific patterns of self-concept development using behavioral and neural measures.

### Self-concept appraisal and domain differences

Self-appraisals can be assessed by examining to what extent adolescents endorse positive and/or negative traits (Rapee *et al.*, 2019). Self-appraisal comprises what we do and how we appear, based on subjective descriptions of our traits (e.g. ‘I am smart’) rather than objective facts (e.g. ‘I have brown eyes’). Children are usually more positive about their subjective traits, describing themselves mainly in positive terms, but positivity about self-traits dips in mid-adolescence (Harter, 2012; van der Aar *et al.*, 2018). These findings fit with prior researches on self-esteem. Self-esteem is defined as the general feeling of self-worth and was previously reported to dip in mid-adolescence (Robins *et al.*, 2002; Robins and Trzesniewski, 2005), but others reported stability during adolescence and increases after mid-adolescence (Orth *et al.*, 2018).

Furthermore, self-appraisals are not necessarily the same across domains or social situations (Harter, 2012; Jankowski *et al.*, 2014). During adolescence, young people need to take on an increasing number of different social roles, in all of which they tend to be a slightly different versions of themselves. They may notice that they have more positive traits in some situations (e.g. social) than others (e.g. academic) (van der Cruijsen *et al.*, 2018). Behavioral research has shown that across development, children increasingly differentiate their self-appraisals across situations or domains (Marsh and Ayotte, 2003; Marsh and Martin, 2011), but it is not known whether or how this process continues throughout adolescence. In this study, we aimed to investigate how self-appraisals differed across age in adolescence and whether these developmental patterns would differ between the academic, physical appearance and prosocial domains.

On the neural level, this study builds on prior fMRI studies in adults that demonstrated consistent activation in the medial prefrontal cortex (mPFC) underlying self-related cues (Denny *et al.*, 2012; Murray *et al.*, 2012), but fewer studies have examined developmental differences in mPFC activation in relation to self-concept development in children and adolescents (e.g. Pfeifer *et al.*, 2009; Pfeifer and Peake, 2012). Some studies including adolescents described stronger mPFC activation underlying self-evaluations in adolescents than in adults (Pfeifer *et al.*, 2007), which fits with a reported quadratic mid-adolescent peak in mPFC activation related to experiencing self-consciousness (Somerville *et al.*, 2013). However, other studies reported no age differences in mPFC activation between adolescents and adults (Jankowski *et al.*, 2014) or during early adolescence (10- to 13-year-old female adolescents) (Barendse *et al.*, 2020). Finally, some studies reported developmental differences in mPFC activity in relation to pubertal hormones (testosterone) only in males but not in females aged 11–14 years (van Buuren *et al.*, 2020).

A few developmental fMRI studies explored domain differences in the development of self-related mPFC activation. For instance, previously reported results from the first time point of the current study (TP1; ages 11–21 years) described linearly increasing mPFC activation associated with physical appearance evaluations (van der Cruijsen *et al.*, 2018). A different, longitudinal study in early adolescents (11–13 years) showed increasing mPFC activation with age and pubertal development for social but not academic self-evaluations (Pfeifer *et al.*, 2013; see also Cosme *et al.*, 2022). Here, we investigated whether mPFC activation underlying self-evaluations would peak in mid-adolescence (Somerville *et al.*, 2013) and whether this developmental pattern would differ between academic, physical and prosocial domains.

### Internalization of perceived peers’ opinions

Social changes in the early teenage years lead adolescents to increasingly value opinions of, and acceptance by, their peers (Felson, 1985; Westenberg *et al.*, 2004; Somerville, 2013). These changes co-occur with improving perspective-taking skills (Dumontheil *et al.*, 2010), which may make adolescents more prone to actually think about opinions that others may have about them (Pfeifer and Peake, 2012). Therefore, the perceived opinions of peers about the self may be an important factor in the developing self-concept. It has indeed been suggested that (perceived) opinions of significant others about the self (reflected self-evaluations; Yeung and Martin, 2003) are important in the construction of one’s own definitions of the self (direct self-evaluations; Felson, 1989; Harter, 2012). In line with this suggestion, a prior study showed that peer rejection becomes internalized in adolescents, resulting in less positive self-views (Rodman *et al.*, 2017). Additionally, we previously demonstrated in a cross-sectional study that the similarity between reflected and direct self-evaluations on the behavioral and neural (mPFC) levels increased in adolescents with increasing age (van der Cruijsen *et al.*, 2019).

In addition to mPFC, temporal–parietal junction (TPJ) is a region implicated especially in reflected versus direct self-processing and is known to be involved in perspective-taking (Schurz *et al.*, 2014). TPJ activation was found to be implicated in both reflected and direct self-evaluations in early adolescents but only in reflected self-evaluations in late adolescents and early adults (Pfeifer *et al.*, 2009; Veroude *et al.*, 2014; van Buuren *et al.*, 2020). Cross-sectional results of the current study sample previously revealed similar TPJ activation for both reflected and direct self-evaluations but found no differences in this pattern of activation with increasing age (van der Cruijsen *et al.*, 2019).

### Current study

**Fig. 1.**
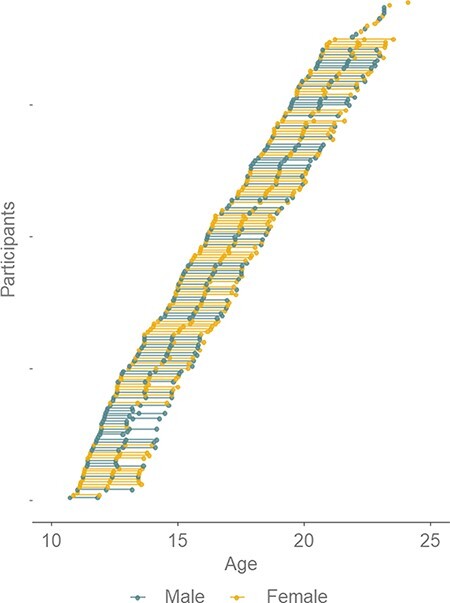
Age distribution of participants at all time points. Dots represent data points, and connected dots represent data points of one participant at different time points. Males are indicated in blue (dark) (*n* = 93) and females in yellow (light) (*n* = 96).

**Fig. 2.**
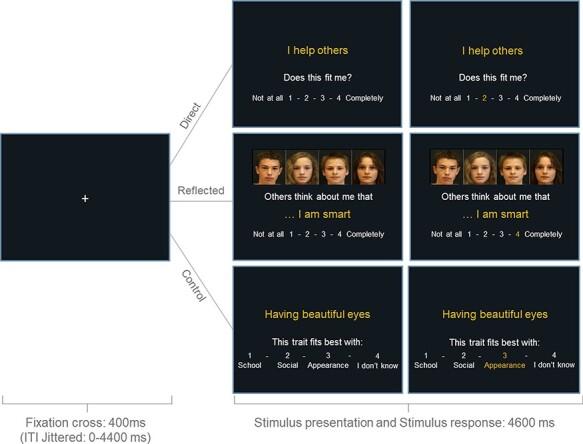
Example of a trial in the direct, reflected and the control condition. Each trial started with a black screen and a jittered duration between 0 and 4400 ms. Subsequently, a fixation cross was shown for 400 ms after which the stimulus appeared. In the direct and reflected conditions, participants rated on a scale of 1 to 4 to what extent the traits described themselves (from their own perspective or their perceived peers’ perspective, respectively). In the control condition, participants categorized the trait sentences into one of the four options. The stimulus was shown for 4600 ms. If participants responded within this timeframe, the number of their choice would turn yellow. If participants failed to respond within this timeframe, a screen with the phrase ‘Too Late!’ was shown for an additional 1000 ms after which the next trial would start.

The current study tested two prevailing theories of self-concept development using a three-wave accelerated longitudinal fMRI design. This approach allowed to capture within-person changes and provides for a more robust test of the behavioral and neural underpinnings of self-concept development. We examined, at the behavioral and neural level, (i) the development of self-appraisal in the academic, physical and prosocial domains and (ii) the development of similarity between reflected and direct self-evaluations. To this end, adolescents and young adults aged 10–24 years (Figure 1) evaluated their traits in the academic, physical and prosocial domains from their own perspective and the perceived perspective of peers in an fMRI task at three annual assessments (Figure 2). The study and hypotheses were pre-registered (https://osf.io/8gc6x/), and deviations from the pre-registered hypotheses are presented in the Supplement.

First, we expected that the positivity of self-appraisals would remain stable until mid-adolescence and increase into late adolescence/young adulthood (Orth *et al.*, 2018) (linear or quadratic age effect). Second, we expected increasing differentiation for self-appraisals between the three domains (Harter, 2012; van der Cruijsen *et al.*, 2018), as demonstrated by different developmental patterns between domains (age × domain interaction). We analyzed positive and negative self-appraisals separately as prior studies reported that adolescents evaluate positive and negative traits differentially in separate domains (van der Aar *et al*., 2018). Indeed, prior studies suggested that positive and negative traits should not necessarily be interpreted as polar opposites, in which the presence of one implies the absence of the other (Bukowski *et al.*, 2018).

On the neural level, we expected a mid-adolescent peak in mPFC activation underlying self-evaluations (Somerville *et al.*, 2013), with increasing differences in mPFC activation for self-evaluations between domains. Based on prior studies, we expected linearly increasing mPFC activation for physical self-evaluations (Van der Cruijsen *et al.*, 2018), no changes in mPFC activation for the academic domain (Van der Cruijsen *et al.*, 2018) and a quadratic peak in mid-adolescence for prosocial self-evaluations (Somerville *et al.*, 2013).

Regarding similarities and differences between perspectives, we expected that behavioral ratings and mPFC activation for reflected and direct self-evaluations would become more similar with age, possibly suggesting more internalization across age (van der Cruijsen *et al.*, 2019). TPJ activation was expected to show increasing differentiation between reflected and direct self-evaluations (Pfeifer *et al.*, 2009; van Buuren *et al.*, 2020). For these hypotheses, we additionally explored domain differences. As pre-registered, we included possible effects of sex in all before-mentioned hypotheses. Given that sex differences were not a primary aim of this study, these findings are presented in the Supplement.

Finally, we tested for longitudinal effects on self-concept outcomes, specifically SCC and fear of negative evaluation (FNE). Previous studies showed that SCC is associated with less susceptibility to external influences such as friends’ behavior or societal ideals (Vartanian and Dey, 2013; Levey *et al.*, 2019). Here, we explored whether these patterns also exist for the positivity of self-evaluations by testing longitudinal relationships between self-evaluations, underlying mPFC activation, and the concepts SCC and FNE.

## Materials and methods

### Participants

At TP1, 160 individuals aged 11–21 years participated in the study of which 10 were excluded due to excessive head movements during the MRI scans (>3 mm total, *n* = 8), not completing the scan (*n* = 1) and a technical error (*n* = 1). The resulting sample consisted of 150 participants (mean age = 15.7, s.d. = 2.9). At TP2, 137 of the initial 160 participants again participated ∼1 year and 1 month later. Additionally, 14 young adolescents (aged 10–12 years, *M *= 11.7, s.d. = 0.49) were included in order to increase sample volume at the left end of our sample. Seven participants were excluded due to excessive head movements during the MRI scans, resulting in a total sample of 144 participants (10–22 years, *M *= 16.9, s.d. = 3.2). At TP3, 130 participants who previously participated at TP1 and/or TP2 again took part in the study ∼1 year and 1 month after TP2. An additional group of 14 adolescents (21–24 years, *M *= 22.85, s.d. = 0.59) were included to increase sample volume at the right end of our sample, and eight participants were excluded from MRI analyses due to excessive head movements during the MRI scans, resulting in a total sample of 136 participants (11–24 years, *M *= 18.7, s.d. = 3.3).

Within the total group of participants (*N* = 189; see Figure 1), 95.2% (*N* = 180) were born in the Netherlands. Participants who were born outside of the Netherlands reported Dutch or European heritage. There were 41 participants with one (*N* = 34) or both (*N* = 7) parents born outside of the Netherlands, of which most were born in other European countries (58%). Parents were asked to report their gross annual family income (11.6% declined to disclose). Fifteen families reported gross annual income <€31 000 (7.9%), whereas 65 families reported an annual income > €76 000 (34.4%); all other families reported middle income.

Intelligence Quotient (IQ) scores were estimated at the TP1 using two subtests of the WISC-III or WAIS-III (similarities and block design). Average IQ scores were above average (*M *= 110.4, s.d. = 11.2), ranging between 80 and 142. IQ did not correlate with age (*r *= 0.016, *P *= 0.828).

All participants and both parents of minors signed informed consent before inclusion in the study, and study procedures were approved by the Medical Ethics Committee of Leiden University Medical Centre.

### Task design and additional measures

#### Task description

The fMRI task (Figure 2) consisted of three separate runs (two experimental runs and one ‘control’ run) in which participants read sentences describing positive and negative traits in academic, physical appearance and prosocial domains. In the experimental runs, participants evaluated to what extent a total of 60 trait sentences (10 positive and 10 negative per domain) described themselves according to their own perspective (direct self-evaluation condition) or the perceived perspective of their peers (reflected self-evaluation condition). In both conditions, participants evaluated the same 60 trait sentences (e.g. ‘I am smart’, ‘I am unattractive’), on a scale of 1 (this trait does not fit me at all) to 4 (this trait completely fits me) by pressing buttons with the index to little finger of their right hand. In the reflected self-condition, the sentences were the same, albeit preceded by the words: ‘Others think about me that…’. Morphed pictures of unknown same-aged peers were shown during this run to remind participants to take their peers’ perspective when evaluating their traits. In the control condition, participants categorized 20 different trait sentences (10 positive and 10 negative) into one of the four categories: (i) school, (ii) social, (iii) appearance or (iv) I do not know.

The three conditions were completed in separate runs, the order of which was counterbalanced between participants. Within each run, trait sentences were presented in a pseudo-randomized order with regard to domains. Trial order was optimized using Optseq (1), which was also used to add jittered intertrial intervals varying between 0 and 4.4 s.

Trials began with a fixation cross (400 ms), whereafter the trait sentence with response options was presented (4600 ms). When participants responded to the stimulus within this timeframe, the number they chose turned yellow for the remaining stimulus time in order to assure participants that their choice had been registered. If participants failed to respond in time, they were shown the phrase ‘Too late!’ for 1000 ms. For TP1, TP2 and TP3, respectively, this occurred on 1.1%, 0.7% and 0.8% of trials in the direct condition; on 1.7%, 1.3% and 1.1% of trials in the reflected condition and on 0.7%, 0.4% and 0.3% of trials in the control condition. These trials were not included in analyses. Intraclass correlation coefficients for direct and reflected self-concept positivity in all domains range between 0.83 and 0.90.

#### SCC

At all time points, participants completed the Dutch version of the SCC scale (Campbell *et al.*, 1996), consisting of 12 items scored on a 5-point scale ranging from 1 (strongly disagree) to 5 (strongly agree). Example items are ‘In general, I have a clear sense of who I am and what I am’ or ‘My beliefs about myself often conflict with one another’ (reverse scored). Mean scores were calculated such that higher scores indicated higher SCC. Cronbach’s alphas for TP1, TP2 and TP3 were *α* = 0.87, *α* = 0.87 and *α* = 0.87, respectively.

#### FNE

At all time points, participants completed the Dutch version of the brief FNE scale (Weeks *et al.*, 2005), consisting of 12 items scored on a 5-point scale ranging from 0 (does not fit me at all) to 4 (fits me very well). Example items are ‘I rarely worry about seeming foolish to others’ (reverse scored) or ‘I worry about what people will think of me even when I know it doesn’t make a difference’. Mean scores were calculated such that higher scores indicated higher FNE. Cronbach’s alphas for TP1, TP2 and TP3 were *α* = 0.96, *α* = 0.97 and *α* = 0.97, respectively.

#### fMRI data acquisition

A Philips 3T MRI scanner with a standard whole-head coil was used to acquire MRI scans. Functional scans were acquired using T2*-weighted echo-planar imaging sequence (TR = 2200 ms, TE = 30 ms, sequential acquisition, 37 slices of 2.75 mm and FOV = 220 × 220 × 111.65 mm). The first two volumes of each run were discarded to account for T1 saturation. After the functional scans, a high-resolution 3D T1-FFE scan for anatomical reference was obtained (TR = shortest ms, TE = 4.6 ms, 140 slices, voxel size = 0.875 mm and FOV = 224 × 178.5 × 168 mm). The task was projected on a screen behind the scanner which the participant could see via a mirror attached to the head coil. Head movement was restricted by placing foam inserts inside the coil.

#### fMRI preprocessing

Data were analyzed using SPM12 (Wellcome Department of Cognitive Neurology, London). Functional scans were corrected for slice-timing acquisition and rigid body movement differences. Structural and functional volumes were spatially normalized to T1 templates by an algorithm using a 12-parameter affine transformation together with a non-linear transformation involving cosine basis functions. The algorithm resampled the volumes to 3 mm cubic voxels. Templates were based on the MNI305 stereotaxic space. Functional volumes were spatially smoothed with a 6 mm FWHM isotropic Gaussian kernel.

Task effects were estimated using the general linear model (GLM) in SPM12. The fMRI data were modeled as a series of zero duration events convolved with the hemodynamic response function. Events of interested modeled were ‘Direct-Academic-Positive’, ‘Direct-Academic-Negative’, ‘Direct-Physical-Positive’, ‘Direct-Physical-Negative’, ‘Direct-Prosocial-Positive’ and ‘Direct-Prosocial-Negative’. The same events were modeled for the reflected condition. For the control condition, only one event of interest was modeled, collapsed across domains and valences. These events were used as covariates in a GLM, together with a basic set of cosine functions that high-pass filtered the data. Six motion regressors were added to the model, and participants who moved >3 mm (1 voxel) were excluded from further analyses. The contrast images computed on a subject-by-subject basis that resulted were submitted to group analyses.

Even after removing participants who moved >3 mm, micro-motion correlated negatively with age at all three time points, indicating that older participants moved less during the scan than younger participants (TP1: *r *= −0.352, *P *< 0.001; TP2: *r *= −.0182, *P *= 0.029; TP3: *r *= −0.058, *P *= 0.499).

### Analyses

#### fMRI contrasts and ROIs

We first performed three whole-brain one-sample *t*-tests for the contrast self > control, applying FDR cluster-level correction (*P *< 0.05) at an initial uncorrected threshold of *P *< 0.001, as implemented in SPM12. Uncorrected *t*-maps can be found on NeuroVault (https://neurovault.org/collections/9265). To investigate our hypotheses regarding mPFC and TPJ activation, we extracted parameter estimates from three regions of interest (ROIs) (8 mm spheres) using the MarsBaR ROI toolbox: mPFC (*x *= −6, *y* = 50, *z* = 4), right TPJ (*x* = −53, *y* = −59, *z* = 20) and left TPJ (*x* = 56, *y* = −56, *z* = 18). ROIs were pre-registered (https://osf.io/8gc6x/) and based on meta-analyses of self-referential processing for mPFC (Denny *et al.*, 2012) and perspective-taking for TPJ (Schurz *et al.*, 2014). Note that ROI sphere radius (8 mm) was not pre-registered but was based on a prior study on our cross-sectional data (van der Cruijsen *et al.*, 2019).

#### Mixed models

Mixed model analyses were performed using the *nlme* package in R (Pinheiro *et al.*, 2020). To test our main pre-registered hypotheses, for each dependent variable (i.e. trait appraisals, mPFC, left TPJ and right TPJ), we fitted a model including the independent variables sex, age (linear and quadratic), perspective (direct, reflected), domain (academic, physical and prosocial), valence (positive and negative) and interactions between these variables. Models included a random intercept of participants to account for variation in starting points. We used multiple comparison correction by dividing the *P*-value of 0.05 by the number of dependent variables, resulting in a *P*-value of 0.05/4 = 0.013. To unpack and interpret interaction effects, we ran follow-up linear mixed models and Tukey *post hoc* tests using the *emmeans* R package (Lenth *et al.*, 2019). To facilitate interpretability of the coefficients and interaction effects, we used sum-to-zero coding [sex was coded as −1 (male) and 1 (female); perspective was coded as −1 (direct) and 1 (reflected); domain was coded as −1, 0 and 1 (for academic, physical and prosocial domains, respectively) and valence was coded as −1 (negative) and 1 (positive)]. The effects of interest to address our main hypotheses are described in the main text, and full model results are presented in Tables 1 and 2. Note that in these full models reported the *F*-tests of all analyses, where quadratic age included linear age as well. *T*-tests regarding individual predictors in the model were conducted in order to determine whether an effect of age was driven by age linear or age quadratic. Because examining effects of sex was a secondary goal, these effects are also presented in the Supplements. Full model results are provided in Tables S1–S4 in the supplementary materials.

**Table 1.** Mixed model statistics for self-evaluations ratings

Behavior: self-appraisals	DFs	*F*-value	*P*-value
*(Intercept)*	*1 (5184)*	*98 213.96*	*0.000*
**Age-quadratic**	**2 (5184)**	**8.33**	**0.000**
**Perspective**	**1 (5184)**	**30.60**	**0.000**
**Domain**	**2 (5184)**	**192.56**	**0.000**
**Valence**	**1 (5184)**	**7586.76**	**0.000**
Perspective×Domain	2 (5184)	0.11	0.897
**Domain**×**Valence**	**2 (5184)**	**121.24**	**0.000**
Perspective×Valence	2 (5184)	3.85	0.050
**Perspective**×**Domain**×**Valence**	**2 (5184)**	**18.17**	**0.000**
Age-quadratic×Perspective	2 (5184)	0.57	0.563
Age-quadratic×Domain	4 (5184)	1.97	0.097
**Age-quadratic**×**Valence**	**2 (5184)**	**30.17**	**0.000**
Age-quadratic×Perspective×Domain	4 (5184)	0.16	0.957
Age-quadratic×Perspective×Valence	2 (5184)	0.83	0.436
**Age-quadratic**×**Domain**×**Valence**	**4 (5184)**	**10.14**	**0.000**
Age-quadratic×Perspective×Domain×Valence	4 (5184)	0.56	0.694

*Note*: Shaded cells indicate the effect did not survive correction for multiple comparisons (at *P *< 0.013).

a Age quadratic includes age linear.

b Full tables including sex are presented in the Supplement.

**Table 2.** Mixed model statistics for neural activity in mPFC, left TPJ and right TPJ ROIs (see text for coordinates)

	DFs	*F*-value	*P*-value
mPFC			
*(Intercept)*	*1 (4906)*	0.01	0.943
**Age-quadratic**	**2 (4906)**	**10.99**	**0.000**
Perspective	1 (4906)	2.04	0.153
**Domain**	**2 (4906)**	**171.66**	**0.000**
**Valence**	**1 (4906)**	**92.82**	**0.000**
Perspective×Domain	2 (4906)	2.07	0.127
Domain×Valence	2 (4906)	2.39	0.092
Perspective×Valence	2 (4906)	2.08	0.149
**Perspective**×**Domain**×**Valence**	**2 (4906)**	**5.32**	**0.005**
**Age-quadratic**×**erspective**	**2 (4906)**	**3.33**	**0.036**
Age-quadratic×Domain	4 (4906)	0.77	0.546
Age-quadratic×Valence	2 (4906)	0.96	0.383
Age-quadratic×Perspective×Domain	4 (4906)	1.05	0.379
Age-quadratic×Perspective×Valence	2 (4906)	0.99	0.370
Age-quadratic×Domain×Valence	4 (4906)	2.08	0.080
Age-quadratic×Perspective×Domain×Valence	4 (4906)	0.09	0.985
Left TPJ			
*(Intercept)*	*1 (4906)*	*36.09*	*0.000*
**Age-quadratic**	**2 (4906)**	**13.26**	**0.000**
**Perspective**	**1 (4906)**	**119.21**	**0.000**
**Domain**	**2 (4906)**	**38.54**	**0.000**
Valence	1 (4906)	0.51	0.474
**Perspective**×**Domain**	**2 (4906)**	**11.20**	**0.000**
**Domain**×**Valence**	**2 (4906)**	**35.93**	**0.000**
Perspective×Valence	2 (4906)	1.52	0.218
Perspective×Domain×Valence	2 (4906)	1.19	0.305
Age-quadratic×Perspective	2 (4906)	0.98	0.377
Age-quadratic×Domain	4 (4906)	0.67	0.611
Age-quadratic×Valence	2 (4906)	2.14	0.118
Age-quadratic×Perspective×Domain	4 (4906)	1.79	0.127
Age-quadratic×Perspective×Valence	2 (4906)	0.73	0.481
Age-quadratic×Domain×Valence	4 (4906)	2.14	0.073
Age-quadratic×Perspective×Domain×Valence	4 (4906)	0.13	0.973
Right TPJ			
*(Intercept)*	*1 (4906)*	*84.93*	*0.000*
**Age-quadratic**	**2 (4906)**	**12.21**	**0.000**
Perspective	1 (4906)	0.99	0.321
**Domain**	**2 (4906)**	**9.24**	**0.000**
Valence	1 (4906)	0.23	0.633
**Perspective**×**Domain**	**2 (4906)**	**9.21**	**0.000**
**Domain**×**Valence**	**2 (4906)**	**9.17**	**0.000**
Perspective×Valence	2 (4906)	0.03	0.873
Perspective×Domain×Valence	2 (4906)	0.19	0.827
Age-quadratic×Perspective	2 (4906)	0.12	0.886
Age-quadratic×Domain	4 (4906)	1.95	0.099
Age-quadratic×Valence	2 (4906)	3.62	0.027
Age-quadratic×Perspective×Domain	4 (4906)	1.45	0.213
Age-quadratic×Perspective×Valence	2 (4906)	0.93	0.394
Age-quadratic×Domain×Valence	4 (4906)	0.34	0.850
Age-quadratic×Perspective×Domain×Valence	4 (4906)	0.84	0.497

*Note:* Shaded cells indicate the effect did not survive correction for multiple comparisons (at *P *< 0.013).

a Age quadratic includes age linear.

b Full tables including sex are presented in the Supplement.

#### Longitudinal prediction analyses

Analyses to test our hypotheses on SCC and FNE were performed in SPSS25. We first tested whether behavioral self-concept appraisal measures at TP1 predicted SCC and FNE at TP2 and TP3. To facilitate interpretation, we calculated a self-concept positivity score from the trait appraisals. For this purpose, the negative items for each domain were reverse scored and together with the ratings on the positive items used to calculate the average positivity. This allowed us to examine whether how positive one is about oneself predicts behavioral outcomes over time.

Additionally, we tested relationships between individual developmental linear slopes for self-positivity (across domains and for domains separately) with individual slopes of SCC and FNE. For this purpose, individual slopes were created in R by fitting a linear age model per variable per participant separately and saving these individual age coefficients (i.e. slopes). These individual slopes were used in subsequent analyses. We controlled for age at TP1. Relationships that did not survive adjusted Bonferroni correction for correlated variables are found in Supplementary Results and in Tables S6–S7 (http://www.quantitativeskills.com/sisa/calculations/bonfer.htm).

## Results

### Behavioral results

#### Self-appraisal

The first analysis examined linear and quadratic age effects on self-concept appraisals including the factors domain, perspective and valence. All statistical effects are found in Table 1. The analysis resulted in main effects of quadratic age, domain, valence, a domain × valence, a quadratic age × valence interaction and a three-way quadratic age × valence × domain interaction.

**Fig. 3.**
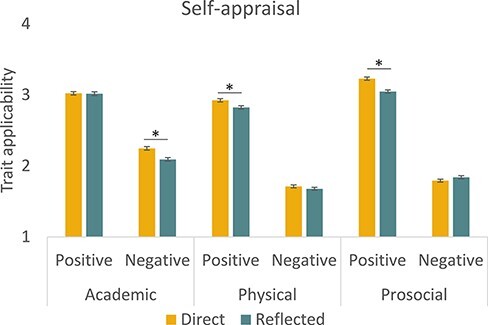
Condition effects for perspective, domain and valence, averaged across time points. Adolescents generally rated their negative academic, positive physical and positive prosocial traits as more applicable to themselves when evaluated from their own perspective as compared to the perceived perspective of their peers. Note that the domain x valence interaction effect depicted here is also encompassed in the age x domain x valence interaction effect depicted in Figure 4.

**Fig. 4.**
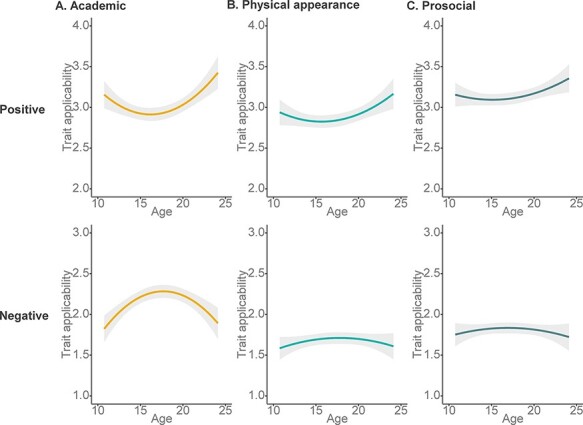
Development of self-concept appraisal across adolescence. Shades indicate 95% confidence intervals. (A) A strong mid-adolescent dip was found in the positivity of the appraisal of academic self-traits. (B) Mid-adolescent dip in the positivity of physical self-concept appraisal. (C) Mid-adolescent dip in the positivity of prosocial self-concept appraisal. (Mid-adolescent dips for (B) and (C) were not significant in *post hoc* testing).

The main effect of valence showed that participants rated negative trials as less applicable than positive trials. The domain × valence interaction revealed that this difference was larger for physical and prosocial than for academic traits (see Figure 3). The quadratic age × valence interaction in self-concept appraisals showed that self-concept ratings for positive traits declined in early-to-mid-adolescence after which ratings increased into late adolescence/early adulthood, with the reversed pattern for negative trait ratings (Figure 4). *Post hoc* analyses confirmed that these effects were observed for both negative traits (mid-adolescent peak) and positive traits (mid-adolescent dip). Finally, the quadratic age × domain × valence interaction revealed the largest self-concept positivity dip for the academic domain specifically, whereas these effects were not statistically significant in *post hoc* tests for physical and prosocial domains (Figure 4A–C).

Our second hypothesis was to test whether self-concept appraisals were different depending on the perspective. Indeed, the analysis resulted in a main effect of perspective, a perspective × valence interaction and a perspective × valence × domain interaction (Figure 3). *Post hoc* comparisons for positive trials showed that direct appraisals were rated more positively than reflected appraisals, but only for physical and prosocial domains, and no difference was found for the academic domain. In contrast, *post hoc* comparisons for negative traits revealed that only for the academic domain, appraisals were rated more negatively from the direct than from the reflected perspective. Thus, perspective effects were dependent on both domain and valence of the traits. None of the perspective effects showed an interaction with age.

### Neural development

#### Whole-brain results.

**Fig. 5.**
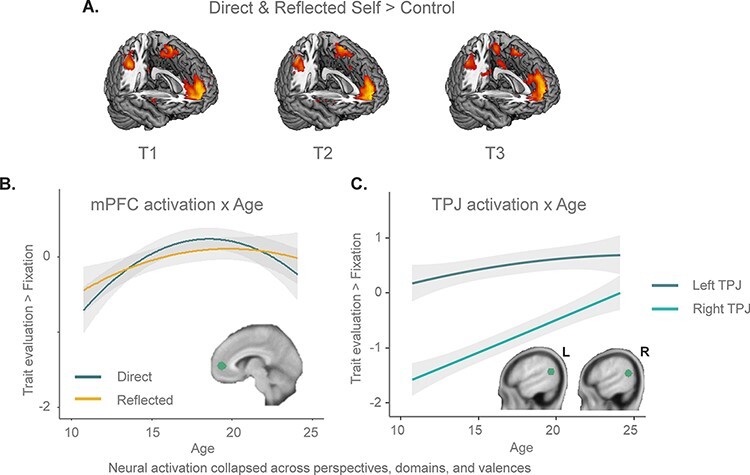
Neural activation underlying direct and reflected self-evaluations. (A) Similar activation pattern in the contrast direct and reflected self > control at the three time points. Common activation in the mPFC, right supramarginal gyrus, PCC and left SMA. (B) Quadratic increase in mPFC activation for direct and reflected self-evaluations across adolescence, leveling off in early adulthood. (C) Linear increase in left and right TPJ activation underlying self-evaluations across adolescence, leveling off in early adulthood. Shades indicate 95% confidence intervals.

Supplementary Table S5 and Figure 5A show results of the whole-brain neural responses to self-evaluations on each time point. Consistent with prior literature, these show activation of mPFC, posterior cingulate cortex (PCC), left supplementary motor area (SMA) and right inferior parietal cortex/supramarginal gyrus in the self > control contrast.

#### ROI mPFC

We extracted the parameter estimates of a pre-registered mPFC ROI, which we used for the linear mixed model analyses and examined domain and valence differences in mPFC development underlying self-evaluations (see Table 2).

First, there was a main effect of quadratic age on mPFC activation relative to fixation baseline. The main quadratic effect of age was best described as increasing activation across adolescence, leveling off in late adolescence (Figure 5B). There was no main effect of perspective, but a quadratic age × perspective interaction (note that this effect did not survive multiple comparison correction). The latter interaction showed a larger peak in mid-adolescence for direct compared to reflected self-appraisals (Figure 5B).

**Fig. 6.**
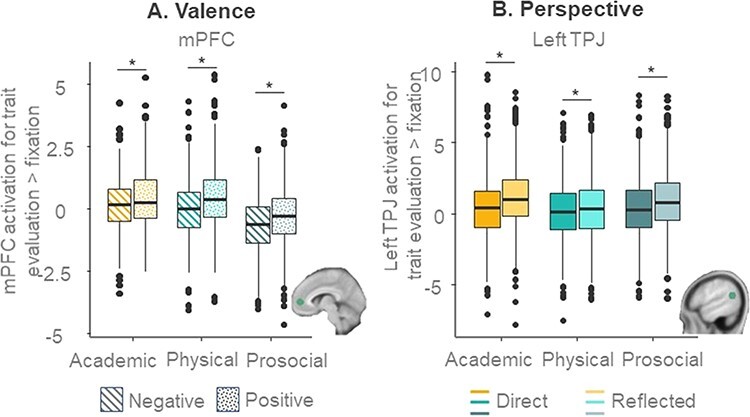
Self-concept appraisal for interactions in mPFC and TPJ. (A) Higher activation for positive than negative trait evaluations in the mPFC, for three domains. (B) Higher activation for reflected than direct trait evaluations in left TPJ, for three domains.

There were significant main effects of domain and valence (Figure 6A). The main effect of valence revealed stronger activity in the mPFC for positive than negative traits. The main effect of domain showed stronger mPFC activation for self-evaluations in the academic and physical appearance domains compared to the prosocial domain.

Finally, we observed a perspective × domain × valence interaction. *Post hoc* analyses for positive and negative traits separately revealed no significant domain × perspective interaction for positive traits. For negative traits, the domain × perspective interaction was significant. Lower activity in the mPFC for prosocial relative to academic and physical traits (as presented in Figure 6A) was observed for both reflected and direct conditions, but for negative traits, the effect was more pronounced in the reflected than the direct condition.

#### ROI TPJ

We extracted the parameter estimates of pre-registered left and right TPJ ROIs to test TPJ activation differentiates between reflected and direct self-evaluations using linear mixed model analyses (see Table 2).

A main effect of age driven by linear age indicated generally stronger left TPJ activation with increasing age (Figure 5C). A main effect of perspective showed that left TPJ activation was higher for reflected compared to direct self-evaluations, but there was no significant age × perspective interaction. A perspective × domain interaction (Figure 6B) revealed that higher activity for reflected relative to direct appraisals was stronger for the academic and prosocial domains than for the physical appearance domain, but the effects were significant in all domains.

For right TPJ, there was no main effect of perspective, but there was a significant perspective × domain interaction, in a similar direction as for left TPJ. Further, similar as for left TPJ, there was main effect of age (driven by linear age) showing increased right TPJ recruitment with age (Figure 5C). There were no interactions between age and perspective for the right TPJ.

Together, the results indicate generally stronger bilateral TPJ activation for reflected compared to direct self-evaluations and overall linear increases in bilateral TPJ activation with age.

### Random effects of age

Although not pre-registered, we tested for random effects of linear age in order to show possible differences in developmental trajectories across time points between participants. We performed log-likelihood ratio tests comparing models with random linear age slopes to models without these slopes separately for the behavioral model and the models regarding mPFC, right and left TPJ. The results indicated that including random effects of linear age into the behavioral model did not improve model fit. However, including random linear age slopes in the mPFC, left and right TPJ models did result in a significantly better fit (all *P-*values < 0.001).

#### Predictions for SCC and FNE

**Fig. 7.**
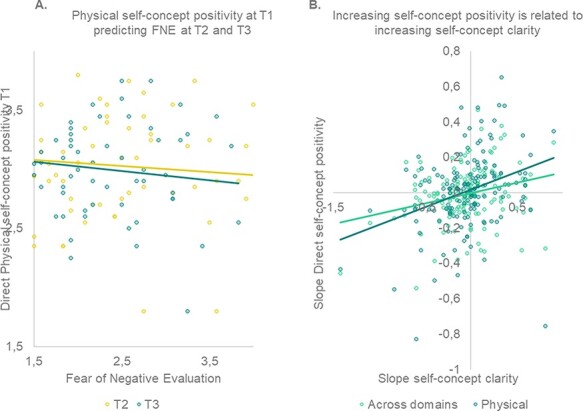
Self-concept appraisal predicting FNE and SCC. (A) Higher appraisal of direct physical self-concept at TP1 predicts less FNE at TP2 and TP3 (i.e. 1 and 2 years later). (B) Increasing direct self-concept appraisal across domains and for the physical domain specifically is related to increasing SCC.

Our final aim was to use longitudinal analyses to examine whether self-concept positivity (reverse scored for negative traits and averaged across valences) predicted behavioral outcomes over time. We found that more positive direct physical self-concept appraisals at TP1 predicted less FNE at TP2 and TP3 (both *P*-values = 0.001; Figure 7A). We also tested whether longitudinal changes in direct self-concept positivity were related to longitudinal changes in SCC. Indeed, greater longitudinal increases in direct self-positivity were related to greater longitudinal increases in SCC (collapsed across domains: *P *= 0.005; physical domain: *P *< 0.001; Figure 7B).

Finally, we found no associations between SCC and FNE with neural activation (all *P*-values > 0.086).

## Discussion

This longitudinal behavioral neuroimaging study provides a comprehensive analysis of self-concept appraisal development in adolescence. By examining evaluation of self-traits and underlying neural activity changes among three developmentally important domains (academic, physical and prosocial), we demonstrated adolescence as a unique developmental window for self-concept development.

First, mid-adolescents demonstrated a dip in self-concept positivity (i.e. endorsements of positive traits and rejection of negative traits). Second, self-evaluations were more positive from a direct relative to reflected perspective for physical appearance and prosocial traits, and more negative from a direct relative to reflected perspective for academic traits, showing domain-specific perspective effects, independent of adolescents’ age. Third, self-appraisals were associated with an increase in mPFC activation, a region typically associated with self-referential processing (Denny *et al.*, 2012; van der Aar *et al.*, 2019). Appraisal of reflected traits was associated with higher TPJ activation, a region typically associated with perspective-taking (Schurz *et al.*, 2014). Fourth, we observed a general monotonic developmental increase in mPFC and bilateral TPJ activation for self-appraisals relative to fixation. For mPFC, the quadratic peak was more pronounced for direct than for reflected self-evaluations. Fifth, higher self-concept appraisal predicted higher SCC and less FNE 1 and 2 years later, showing that self-appraisal development is formative for developing a stable and confidently defined self.

### Self-concept development and domain differences

Our first aim was to examine self-concept development in three domains that are relevant for adolescent development: the academic domain given that this is a major performance area for adolescents (Wehrens *et al.*, 2010; Marsh and Martin, 2011), the physical domain given the large changes in physical appearance and social comparisons (Blakemore *et al.*, 2010) and finally the prosocial domain given that adolescence a period of transition from self to other-oriented behavior (Fuligni, 2019). As predicted, we observed mid-adolescent dips in positivity of self-concept appraisal. This may reflect higher identity uncertainty in this period (Becht *et al.*, 2016). Some prior studies suggested that a period of uncertainty can be adaptive for developing a coherent self-concept (Erikson, 1968). That is, questioning the self may eventually lead to better-suited or more appropriate views of the self.

When comparing domains, we observed that the mid-adolescent dip in self-concept positivity was most pronounced in the academic domain, relative to physical and prosocial domains. One explanation could be that this is related to school transitions or choosing study directions in the second phase of Dutch high school, where adolescents are possibly more aware of their performance relative to classmates (Eccles *et al.*, 1993; Schaffhuser *et al.*, 2017). Alternatively, the differences in domain-specific patterns may indicate the largest domain differentiation in mid-adolescence which levels off in late adolescence and early adulthood. This would be consistent with Harter’s view that across adolescent development domains become more differentiated with increasing age, whereas consistency of self-concept increases in young adulthood (Sebastian *et al.*, 2008; Harter, 2012). Finally, in the Supplementary Material, we report that females showed a larger mid-adolescent dip in self-concept positivity for physical appearance traits relative to males. These findings show that biological (Van Buuren *et al.*, 2020) or socialization experiences (Van der Meulen *et al.*, 2017) may affect adolescents in gender-specific ways, but this question should be addressed in more detail in future studies.

### Reflected and direct self-concept

The second main question addressed in this study concerned the developmental patterns of reflected self-concept. It has been theorized that an important aspect of the developing self-concept is to integrate perspectives of self and others (Gecas, 1982; Felson, 1985). Our results indicated that adolescents are generally more positive about themselves from the direct than the reflected perspective, except for the academic domain. Development of self-evaluations from both perspectives is similar, suggesting that internalization of direct and reflected perspectives of the self emerges largely before adolescence, possibly in childhood (Harter, 2012).

### Neural activation underlying self-evaluation

The whole-brain results are consistent with a large set of fMRI studies showing higher activity in the mPFC for self-appraisals relative to a control condition (Denny *et al.*, 2012; Veroude *et al*., 2014). Additional results showed that activation in the mPFC was stronger for evaluation of positive compared to negative traits, and negative traits were often rejected whereas positive traits were more often endorsed. mPFC activation therefore proposed reflects self-relevance or personal significance (Moran *et al.*, 2006; D’Argembeau, 2013).

In terms of age-related patterns, we observed a general quadratic age pattern of self-related mPFC activation (relative to fixation baseline) during adolescence and leveling off in late adolescence, as well as a differential adolescent peak depending on the perspective. Specifically, we observed a larger mid-adolescent peak for direct than for reflected self-appraisals and more similarity between direct and reflected self-appraisals in older adolescence. These findings are consistent with a prior study showing heightened self-consciousness-related mPFC activation in mid-adolescence (Somerville *et al.*, 2013). The larger early-adolescence difference between reflected and direct self-appraisals adds to our prior work showing that with increasing age, mPFC activation for reflected and direct self-appraisals become more aligned (van der Cruijsen *et al.*, 2019).

Left TPJ activation (involved in third-person perspective-taking; Schurz *et al.*, 2014) was stronger for reflected versus direct self-evaluations. This pattern did not change with age, indicating that across adolescence, individuals engage more in third-person perspective-taking when evaluating themselves from the perceived perspective of peers compared to doing so from their own perspective. This is interesting as stronger TPJ activation for reflected versus direct self-evaluations was previously found in late adolescents and early adults (Pfeifer *et al.*, 2009; Veroude *et al.*, 2014), but not in young adolescents (Pfeifer *et al.*, 2009). TPJ activation across both perspectives (relative to fixation) did increase across adolescence, suggesting that neural activity in this region increases similarly for both reflected and direct self-evaluations, underlining adolescence as a sensitive window during which opinions of both self and peers are similarly important in self-evaluations.

Even though mPFC and TPJ were sensitive to domains (see also Cosme *et al.*, 2022), the developmental pattern of mPFC and TPJ activation underlying self-evaluations did not differ between domains, suggesting that mPFC serves as a general mechanism for thinking about self across domains, at least for the domains that were included in this study. These findings are not consistent with our initial predictions and may therefore be sample or study specific. A prior study that included social traits observed differential age patterns in the mPFC for social versus academic domains (Cosme *et al.*, 2022; but see Jankowski *et al.*, 2014 for an absence of domain effects in the mPFC); therefore, developmental patterns may be specific for social relative to prosocial domains (see also Blakemore and Mills, 2014).

### SCC and FNE

A final important question is whether the longitudinal design of this study allowed us to predict coherence of self-concept and confidential self-evaluations over time (Harter, 2012; Schwartz *et al.*, 2012). Indeed, we showed that negative physical appearance self-appraisal is predictive of FNE and that increases in physical self-concept appraisal over time were related to increasing SCC. FNE was previously linked to eating disorders (Trompeter *et al.*, 2019) and low SCC to negative body image, internalizing societal attractiveness standards and appearance-related social comparisons (Vartanian and Dey, 2013; Vartanian *et al.*, 2016; Seo et al., 2020). Therefore, we suggest that promoting positivity of physical self-appraisal can be beneficial for developing a clear and positive self-concept. Specifically, positive physical self-concept may provide adolescents with the confidence to accept themselves as they are (i.e. to feel less fear for negative evaluation by others) and with a clear concept of self.

The degree of similarity between reflected and direct self-evaluations was not robustly related to SCC or FNE 1 and 2 years later. Thus, we could not confirm a predictive relationship between reflected- and direct-self similarity with outcome measures. Additionally, mPFC activation for self-evaluations was not predictive of future SCC or FNE, suggesting that brain activation in this region is a correlate of behavioral processes rather than a predictor of future psychological constructs.

### Future directions: strengths and limitations

Strengths of the current study are the use of multiple methods to measure self-concept in different domains, the even distribution of participants aged between 10 and 24 years and the use of an accelerated longitudinal design including up to three waves of data within participants. Advantages of this design are (i) the possibility to estimate trajectories on a group level with more power due to multiple measurements in each participant and (ii) the opportunity to test whether the current measures can predict future outcomes. Several findings result in open questions for future research. Specifically, random effects of age were observed for neural activity in the mPFC and TPJ relative to fixation. Therefore, in order to fully understand the development of self-concept domain differentiation in adolescence, future research should aim to investigate domain differentiation in on a within-person level.

Future studies would also benefit from specific task or sample adaptations. First, although the current study included evaluations of adaptive social traits (i.e. prosocial behavior), it would be interesting to include in future studies a more generally social domain targeted toward more daily peer interactions, for example, by including traits such as ‘I am kind’ or ‘I am shy’ (Cosme *et al.*, 2022). Second, the reflected condition presented faces, whereas the direct condition did not. A prior study did not observe significant differences in brain activation for reflected and direct appraisals (van der Cruijsen *et al.*, 2019), suggesting that participants engaged a similar brain network. However, future studies should include better control conditions, for example, using blurred faces (Van Hoorn *et al.*, 2016). Third, participants in this study had a relatively high socioeconomic status background, which should be kept in mind when interpreting the results.

## Conclusion

This study provides a novel perspective to a unique window in adolescent development by demonstrating dissociable behavioral and neural patterns for self-concept development across domains and perspectives. Self-concept appraisal showed a peak in mid-adolescent sensitivity and uncertainty. Reflected and direct self-evaluations were domain specific, emphasizing that adolescence is a sensitive phase during which perceived opinions of others are increasingly important in self-evaluations. These patterns were associated with domain- and perspective-dependent differences in activation in the mPFC and TPJ. Self-concept positivity predicted less future FNE, verifying that having a positive sense of self is beneficial on multiple levels. Together, this study confirms adolescence as an important formative phase for developing a suitable and positive concept of self.

## Supplementary Material

nsac062_Supp

## Data Availability

Data, protocols and materials used in the current study can be accessed on request by contacting the first author of this paper. This study is part of the Leiden Self-Concept study, of which data from the first time point have previously been reported (Van der Cruijsen *et al.*, 2018; van der Cruijsen *et al.*, 2019).
