# Supplementary material for: Longitudinal self-concept development in adolescence
Source: Soc Cogn Affect Neurosci. 2023 Jan 14;18(1):nsac062. doi: 10.1093/scan/nsac062 (PMC10036877; doi:10.1093/scan/nsac062)
Supplement: nsac062_Supp [file nsac062_supp.zip › scan-22-038-File002.docx]

**Supplementary Information Text**

**SI Results**

**Sex differences in self-appraisals**

An additional goal of this study was to examine sex differences in self-appraisals. The model indicated a 4-way interaction effect including sex, quadratic age, domain and valence (p<.001), Supplementary Table 1. Follow-up linear mixed models per valence separately revealed a significant sex x age x domain interaction effect for positive traits only (F(4, 6.89), p<.001; negative p = .10). Subsequent follow-up analyses for each domain separately showed that in the physical domain only, there was a significant sex x age quadratic interaction effect (F(2, 709) = 3.05, p = .048) (this interaction did not survive multiple comparison correction), qualified by a mid-adolescent dip in positive physical trait application in girls (see Supplemental Figure 1B).

**Sex differences in neural activation**

We next aimed to examine sex differences in the neural activation underlying the evaluation of self-traits.

***mPFC.*** The model including mPFC activation revealed a sex x quadratic age and a sex x perspective interaction effect (both *p*-values <.001; Supplementary Table 2). The sex x age interaction effect was qualified by a significantly larger increase in activation with age in females (*b*=.069, *SE*=.019, *p*<.001) (Supplemental Figure 2A). A post-hoc test on the significant sex x perspective interaction revealed that for females, mPFC activation was significantly stronger for reflected compared to direct self-evaluations (*b*=.233, *SE*=.07, *p*=.001).

***TPJ.*** The models including left and right TPJ activation both revealed a sex x age (left TPJ *p*<.001; right TPJ *p*=.008), and a sex x perspective (left TPJ *p*<.001; right TPJ; *p*=.002) interaction effect (see Supplementary Table 3 and 4. Both sex x age interaction effects were qualified by stability in TPJ activation across adolescence for males (left TPJ: *p*=.58; right TPJ: *p*=.20), and a linear increase in activation with age in females (left TPJ: *b*=.18, *SE*=.027, *p*=<.001; right TPJ: *b*=.14, *SE*=.025, *p*<.001) (Supplemental Figure 2B,C).

In left TPJ, the sex x perspective interaction effect showed that for males, left TPJ activation was higher for reflected compared to direct self-evaluations (males: b=-.72, SE=.09, p<.001; females: b=-.15, SE=.09, p=.081) (Supplementary Figure 3A). In contrast, the sex x perspective interaction effect for right TPJ showed that activation in this region was stronger for direct compared to reflected self-evaluations in females (males: *b*=-.14, *SE*=.08, *p*=.10; females: *b*=.21, *SE*=.08, *p*=.006) (Supplementary Figure 3B).

**Relationships of self-concept with behavior.**

Relationships that did survived adjusted Bonferroni correction for correlated variables (<http://www.quantitativeskills.com/sisa/calculations/bonfer.htm>) are marked with an asterisk (*).

Direct self-concept positivity at T1 predicted self-concept clarity at T2 and T3 (T2: *p*=.011; T3: *p*=.015), and both direct (negatively) and reflected (positively) self-concept positivity at T1 predicted fear of negative evaluation at T3 (Direct: *p*=.042; Reflected: *p*=.041). Regressions for the domains separately showed that self-concept clarity at T2 and T3 was predicted by direct (T2: *p*=.033; T3: *p*=.020) and reflected (T2: *p*=.025; T3: *p*=.018) academic self-concept positivity. Direct physical self-concept additionally predicted self-concept clarity at T2 (*p*=.016). Fear of negative evaluation at T2 and T3 was predicted by direct (T2: *p*=.001*; T3: *p*=.001*) and reflected (T2: *p*=.003; T3: *p*=.016) physical self-concept at T1, and Reflected academic self-concept additionally predicted fear of negative evaluation at T3 (*p*=.009).

Across domains, the difference between reflected minus direct self-concept positivity at T1 predicted fear of negative evaluation at T3 (*p*=.038). Regressions for the domains separately showed that the difference score in the academic domain predicted self-concept clarity at T2 (*p*=.023) and T3 (*p*=.022). Fear of negative evaluation at T2 and T3 was predicted by the self-concept difference score in the physical domain (T2: *p*=.013; T3: *p*=.033). The difference score in the academic domain additionally predicted fear of negative evaluation at T3 (*p*=.027).

Next, we extracted individual developmental slopes for the behavioral measures of self-concept and self-concept clarity and fear of negative evaluation. These analyses revealed positive relationships between the slopes of direct self-concept positivity (*p*=.005*), and of direct physical self-concept positivity (*p*<.001*) with the slope of SCC (see also main text). The difference between reflected and direct self-positivity (*p*=.047), was negatively related to the slope of SCC.

***Neural relationships.*** None of the relationships between mPFC activation at T1 and SCC or FNE at T2 or T3 were significant (see Supplementary Table S6). The individual developmental slope of mPFC activation for reflected prosocial self-evaluations was positively related to fear of negative evaluation at T3 (*p*=.011).

**Supplementary Figures:**


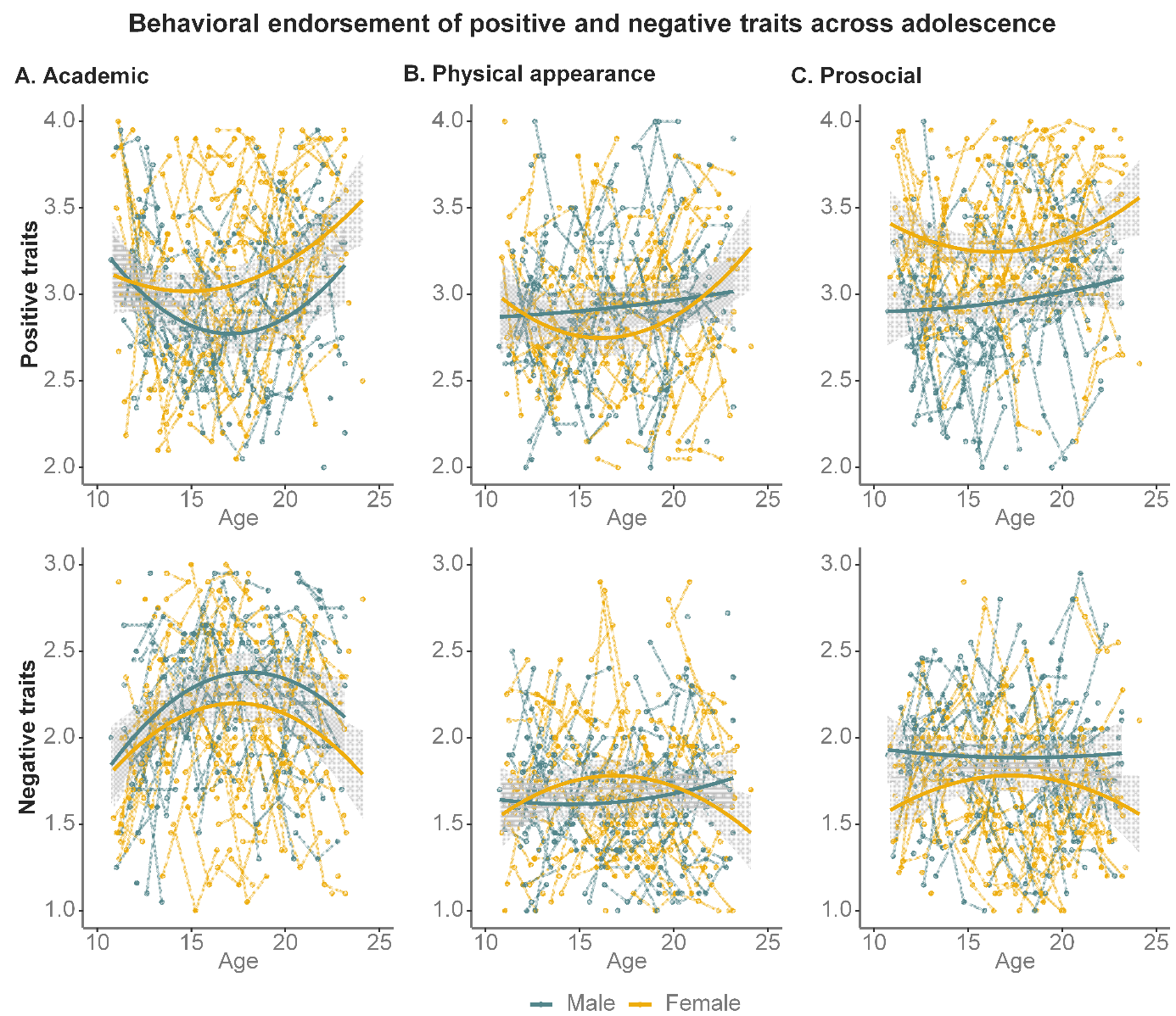


**Figure S1**. Development of endorsement of traits, separated by positive and negative traits, across adolescence, plotted for males and females separately. Shades indicate 95% confidence intervals. *A.* A strong mid-adolescent *dip* was found in endorsement of *positive* academic self-traits, and a strong mid-adolescent *peak* was found in endorsement of *negative* academic self-traits. *B.* Mid-adolescent dip in positive and peak in negative physical trait-endorsements in females, but not in males. *C*. No changes in the endorsement of positive and negative prosocial traits with age.


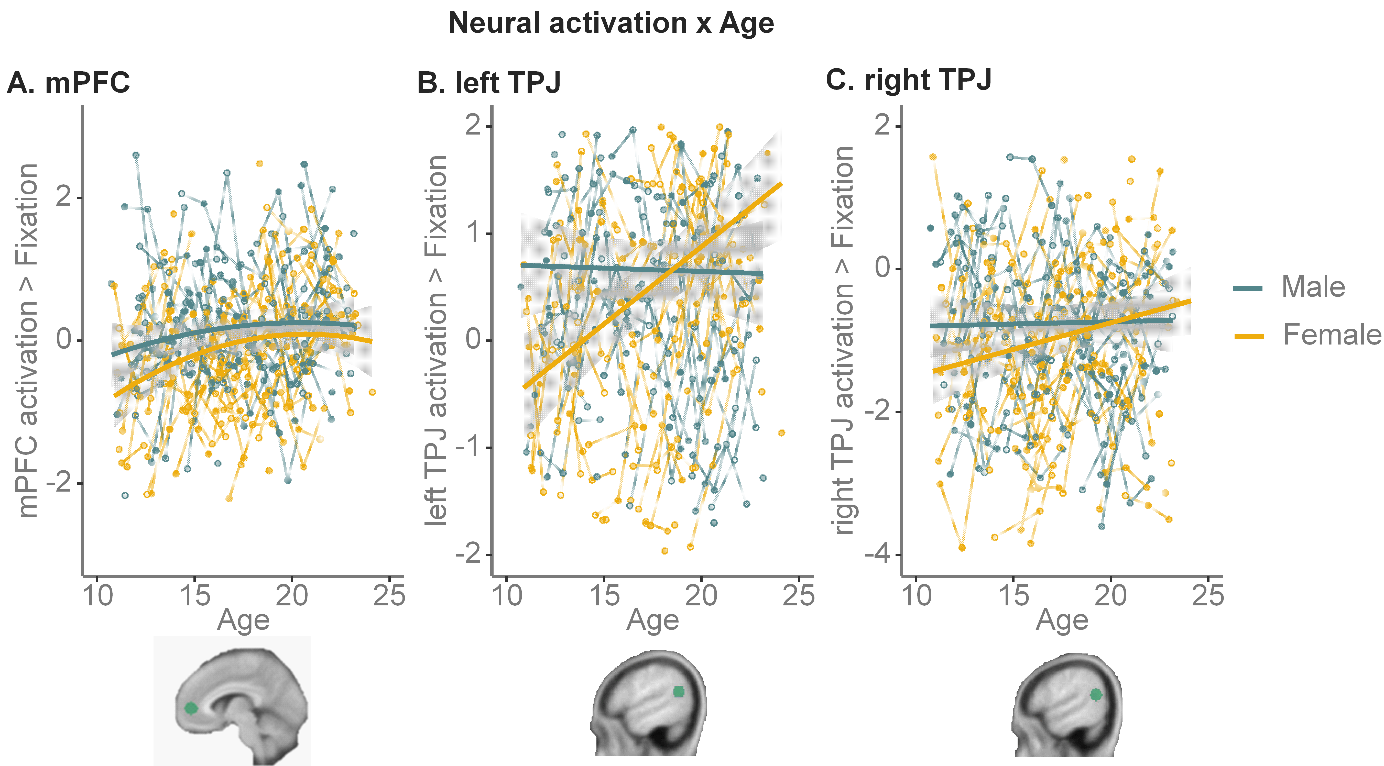
**Figure S2.** Development of mPFC and TPJ activity across adolescence, plotted for males and females separately. Shades indicate 95% confidence intervals.


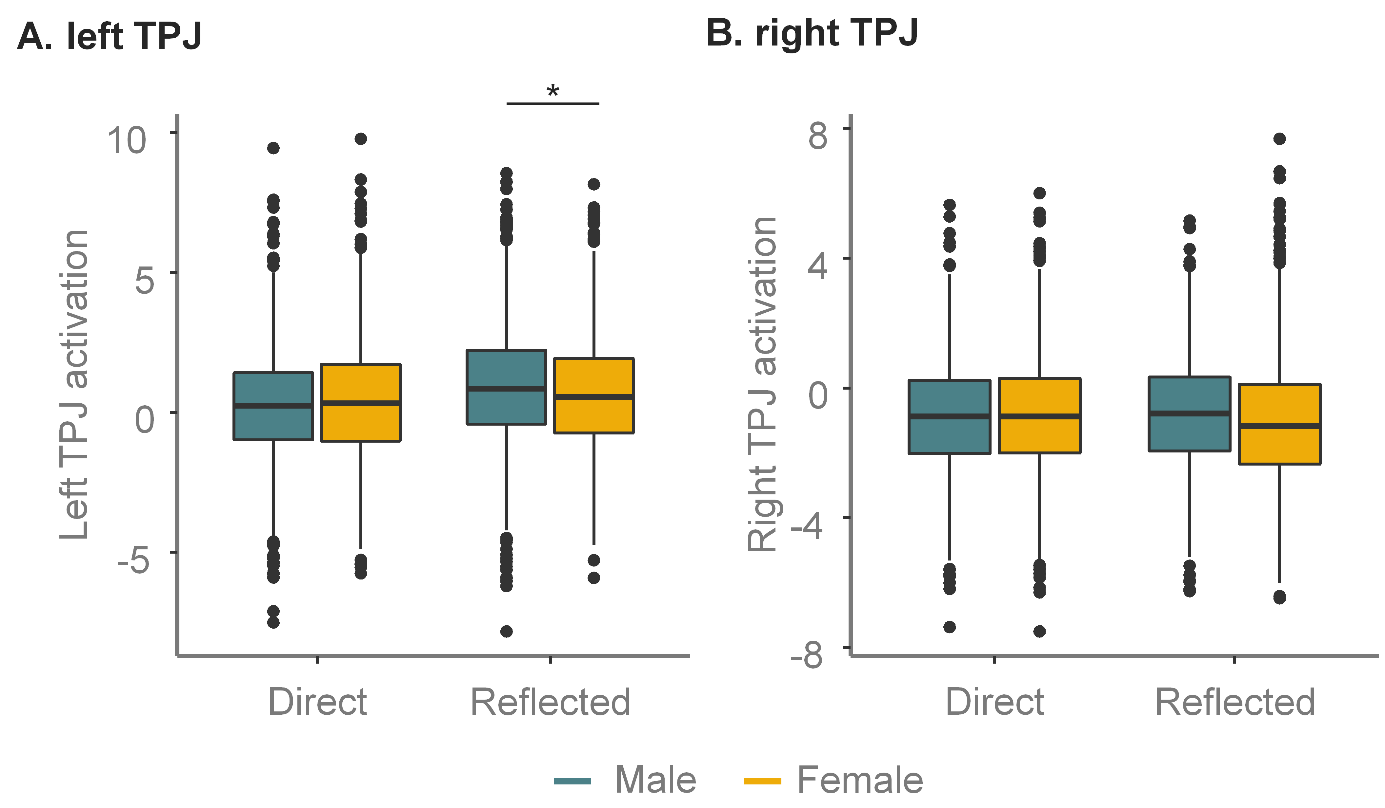


**Figure S3.** Left and right TPJ activation for direct and reflected conditions, separated for males and females. Males showed higher left TPJ activity for reflected than direct trials compared to females.

**Table S1**

Mixed model statistics: Self-appraisals

| Self-Appraisals |  |  |  |
| --- | --- | --- | --- |
|  | df | F-value | p-value |
| (Intercept) | 1 (5184) | 98213.96 | 0.000 |
| Sex | 1 (186) | 4.95 | 0.027 |
| Age-quadratic | 2 (5184) | 8.33 | 0.000 |
| Perspective | 1 (5184) | 30.60 | 0.000 |
| Domain | 2 (5184) | 192.56 | 0.000 |
| Valence | 1 (5184) | 7586.76 | 0.000 |
| Sex*Age-quadratic | 2 (5184) | 0.33 | 0.716 |
| Sex*Perspective | 1 (5184) | 0.01 | 0.917 |
| Age-quadratic*Perspective | 2 (5184) | 0.57 | 0.563 |
| Sex*Domain | 2 (5184) | 4.60 | 0.010 |
| Age-quadratic*Domain | 4 (5184) | 1.97 | 0.097 |
| Perspective*Domain | 2 (5184) | 0.11 | 0.897 |
| Sex*Valence | 1 (5184) | 72.84 | 0.000 |
| Age-quadratic*Valence | 2 (5184) | 30.17 | 0.000 |
| Perspective*Valence | 1 (5184) | 3.85 | 0.050 |
| Domain*Valence | 2 (5184) | 121.24 | 0.000 |
| Sex*Age-quadratic*Perspective | 2 (5184) | 0.41 | 0.667 |
| Sex*Age-quadratic*Domain | 4 (5184) | 0.92 | 0.451 |
| Sex*Perspective*Domain | 2 (5184) | 0.27 | 0.762 |
| Age-quadratic*Perspective*Domain | 4 (5184) | 0.16 | 0.957 |
| Sex*Age-quadratic*Valence | 2 (5184) | 4.35 | 0.013 |
| Sex*Perspective*Valence | 1 (5184) | 3.75 | 0.053 |
| Age-quadratic*Perspective*Valence | 2 (5184) | 0.83 | 0.436 |
| Sex*Domain*Valence | 2 (5184) | 57.56 | 0.000 |
| Age-quadratic*Domain*Valence | 4 (5184) | 10.14 | 0.000 |
| Perspective*Domain*Valence | 2 (5184) | 18.17 | 0.000 |
| Sex*Age-quadratic*Perspective*Domain | 4 (5184) | 0.07 | 0.990 |
| Sex*Age-quadratic*Perspective*Valence | 2 (5184) | 1.84 | 0.159 |
| Sex*Age-quadratic*Domain*Valence | 4 (5184) | 5.64 | 0.000 |
| Sex*Perspective*Domain*Valence | 2 (5184) | 1.45 | 0.234 |
| Age-quadratic*Perspective*Domain*Valence | 4 (5184) | 0.56 | 0.694 |
| Sex*Age-quadratic*Perspective*Domain*Valence | 4 (5184) | 0.67 | 0.615 |

*** Age quadratic includes Age linear**

**Table S2**

Mixed model statistics: mPFC activation

| mPFC activation |  |  |  |
| --- | --- | --- | --- |
|  | numDF | F-value | p-value |
| (Intercept) | 1 (4906) | 36.09 | 0.000 |
| Sex | 1 (182) | 1.07 | 0.301 |
| Age-quadratic | 2 (4906) | 13.26 | 0.000 |
| Perspective | 1 (4906) | 119.21 | 0.000 |
| Domain | 2 (4906) | 38.54 | 0.000 |
| Valence | 1 (4906) | 0.51 | 0.474 |
| Sex*Age-quadratic | 2 (4906) | 9.42 | 0.000 |
| Sex*Perspective | 1 (4906) | 20.00 | 0.000 |
| Age-quadratic*Perspective | 2 (4906) | 0.98 | 0.377 |
| Sex*Domain | 2 (4906) | 2.17 | 0.114 |
| Age-quadratic*Domain | 4 (4906) | 0.67 | 0.611 |
| Perspective*Domain | 2 (4906) | 11.20 | 0.000 |
| Sex*Valence | 1 (4906) | 0.54 | 0.464 |
| Age-quadratic*Valence | 2 (4906) | 2.14 | 0.118 |
| Perspective*Valence | 1 (4906) | 1.52 | 0.218 |
| Domain*Valence | 2 (4906) | 35.93 | 0.000 |
| Sex*Age-quadratic*Perspective | 2 (4906) | 2.75 | 0.064 |
| Sex*Age-quadratic*Domain | 4 (4906) | 1.02 | 0.394 |
| Sex*Perspective*Domain | 2 (4906) | 0.14 | 0.869 |
| Age-quadratic*Perspective*Domain | 4 (4906) | 1.79 | 0.127 |
| Sex*Age-quadratic*Valence | 2 (4906) | 0.56 | 0.572 |
| Sex*Perspective*Valence | 1 (4906) | 4.07 | 0.044 |
| Age-quadratic*Perspective*Valence | 2 (4906) | 0.73 | 0.481 |
| Sex*Domain*Valence | 2 (4906) | 3.07 | 0.047 |
| Age-quadratic*Domain*Valence | 4 (4906) | 2.14 | 0.073 |
| Perspective*Domain*Valence | 2 (4906) | 1.19 | 0.305 |
| Sex*Age-quadratic*Perspective*Domain | 4 (4906) | 1.31 | 0.263 |
| Sex*Age-quadratic*Perspective*Valence | 2 (4906) | 0.19 | 0.831 |
| Sex*Age-quadratic*Domain*Valence | 4 (4906) | 0.51 | 0.725 |
| Sex*Perspective*Domain*Valence | 2 (4906) | 1.93 | 0.145 |
| Age-quadratic*Perspective*Domain*Valence | 4 (4906) | 0.13 | 0.973 |
| Sex*Age-quadratic*Perspective*Domain*Valence | 4 (4906) | 0.59 | 0.666 |

*** Age quadratic includes Age linear**

**Table S3**

Mixed model statistics: left TPJ activation

| Left TPJ activation |  |  |  |
| --- | --- | --- | --- |
|  | numDF | F-value | p-value |
| (Intercept) | 1 (4906) | 36.09 | 0.000 |
| Sex | 1 (182) | 1.07 | 0.301 |
| Age-quadratic | 2 (4906) | 13.26 | 0.000 |
| Perspective | 1 (4906) | 119.21 | 0.000 |
| Domain | 2 (4906) | 38.54 | 0.000 |
| Valence | 1 (4906) | 0.51 | 0.474 |
| Sex*Age-quadratic | 2 (4906) | 9.42 | 0.000 |
| Sex*Perspective | 1 (4906) | 20.00 | 0.000 |
| Age-quadratic*Perspective | 2 (4906) | 0.98 | 0.377 |
| Sex*Domain | 2 (4906) | 2.17 | 0.114 |
| Age-quadratic*Domain | 4 (4906) | 0.67 | 0.611 |
| Perspective*Domain | 2 (4906) | 11.20 | 0.000 |
| Sex*Valence | 1 (4906) | 0.54 | 0.464 |
| Age-quadratic*Valence | 2 (4906) | 2.14 | 0.118 |
| Perspective*Valence | 1 (4906) | 1.52 | 0.218 |
| Domain*Valence | 2 (4906) | 35.93 | 0.000 |
| Sex*Age-quadratic*Perspective | 2 (4906) | 2.75 | 0.064 |
| Sex*Age-quadratic*Domain | 4 (4906) | 1.02 | 0.394 |
| Sex*Perspective*Domain | 2 (4906) | 0.14 | 0.869 |
| Age-quadratic*Perspective*Domain | 4 (4906) | 1.79 | 0.127 |
| Sex*Age-quadratic*Valence | 2 (4906) | 0.56 | 0.572 |
| Sex*Perspective*Valence | 1 (4906) | 4.07 | 0.044 |
| Age-quadratic*Perspective*Valence | 2 (4906) | 0.73 | 0.481 |
| Sex*Domain*Valence | 2 (4906) | 3.07 | 0.047 |
| Age-quadratic*Domain*Valence | 4 (4906) | 2.14 | 0.073 |
| Perspective*Domain*Valence | 2 (4906) | 1.19 | 0.305 |
| Sex*Age-quadratic*Perspective*Domain | 4 (4906) | 1.31 | 0.263 |
| Sex*Age-quadratic*Perspective*Valence | 2 (4906) | 0.19 | 0.831 |
| Sex*Age-quadratic*Domain*Valence | 4 (4906) | 0.51 | 0.725 |
| Sex*Perspective*Domain*Valence | 2 (4906) | 1.93 | 0.145 |
| Age-quadratic*Perspective*Domain*Valence | 4 (4906) | 0.13 | 0.973 |
| Sex*Age-quadratic*Perspective*Domain*Valence | 4 (4906) | 0.59 | 0.666 |

*** Age quadratic includes Age linear**

**Table S4**

Mixed model statistics: right TPJ activation

| Right TPJ activation |  |  |  |
| --- | --- | --- | --- |
|  | numDF | F-value | p-value |
| (Intercept) | 1 (4906) | 84.93 | 0.000 |
| Sex | 1 (182) | 1.34 | 0.248 |
| Age-quadratic | 2 (4906) | 12.21 | 0.000 |
| Perspective | 1 (4906) | 0.99 | 0.321 |
| Domain | 2 (4906) | 9.24 | 0.000 |
| Valence | 1 (4906) | 0.23 | 0.633 |
| Sex*Age-quadratic | 2 (4906) | 4.84 | 0.008 |
| Sex*Perspective | 1 (4906) | 9.60 | 0.002 |
| Age-quadratic*Perspective | 2 (4906) | 0.12 | 0.886 |
| Sex*Domain | 2 (4906) | 4.76 | 0.009 |
| Age-quadratic*Domain | 4 (4906) | 1.95 | 0.099 |
| Perspective*Domain | 2 (4906) | 9.21 | 0.000 |
| Sex*Valence | 1 (4906) | 0.09 | 0.762 |
| Age-quadratic*Valence | 2 (4906) | 3.62 | 0.027 |
| Perspective*Valence | 1 (4906) | 0.03 | 0.873 |
| Domain*Valence | 2 (4906) | 9.17 | 0.000 |
| Sex*Age-quadratic*Perspective | 2 (4906) | 2.86 | 0.057 |
| Sex*Age-quadratic*Domain | 4 (4906) | 0.32 | 0.868 |
| Sex*Perspective*Domain | 2 (4906) | 0.49 | 0.615 |
| Age-quadratic*Perspective*Domain | 4 (4906) | 1.45 | 0.213 |
| Sex*Age-quadratic*Valence | 2 (4906) | 0.24 | 0.790 |
| Sex*Perspective*Valence | 1 (4906) | 2.20 | 0.138 |
| Age-quadratic*Perspective*Valence | 2 (4906) | 0.93 | 0.394 |
| Sex*Domain*Valence | 2 (4906) | 1.54 | 0.214 |
| Age-quadratic*Domain*Valence | 4 (4906) | 0.34 | 0.850 |
| Perspective*Domain*Valence | 2 (4906) | 0.19 | 0.827 |
| Sex*Age-quadratic*Perspective*Domain | 4 (4906) | 0.74 | 0.562 |
| Sex*Age-quadratic*Perspective*Valence | 2 (4906) | 0.10 | 0.903 |
| Sex*Age-quadratic*Domain*Valence | 4 (4906) | 0.10 | 0.982 |
| Sex*Perspective*Domain*Valence | 2 (4906) | 2.83 | 0.059 |
| Age-quadratic*Perspective*Domain*Valence | 4 (4906) | 0.84 | 0.497 |
| Sex*Age-quadratic*Perspective*Domain*Valence | 4 (4906) | 0.61 | 0.656 |

*** Age quadratic includes Age linear**

**Table S5**

*Regions activated for the conjunction of Direct & Reflected Self > Control*

|  |  |  | Timepoint 1 | | | | |  |  | Timepoint 2 | | | |  | Timepoint 3 | | | | |
| --- | --- | --- | --- | --- | --- | --- | --- | --- | --- | --- | --- | --- | --- | --- | --- | --- | --- | --- | --- |
|  | *Region* | *BA* | *Coordinates* | | | *Cluster Size* | *T* |  | *Coordinates* | | | *Cluster Size* | *T* |  | *Coordinates* | | | *Cluster Size* | *T* |
| Frontal/ Subcortical | R Superior Medial Frontal (mPFC) | 10/9 | 4 | 60 | 14 | 3815 | 7.17 |  | -6 | 56 | 4 | 2133 | 7.45 |  | 2 | 62 | 10 | 3134 | 8.40 |
|  |  |  | -10 | 50 | -4 |  | 6.64 |  | 4 | 46 | 20 |  | 7.43 |  | 2 | 56 | 24 |  | 8.03 |
|  |  |  | 44 | 42 | 14 |  | 6.49 |  | 4 | 60 | 4 |  | 7.07 |  | -22 | 42 | 26 |  | 7.12 |
|  | R Inferior Frontal Operc. | 44 | 54 | 8 | 20 | 230 | 5.80 |  | 50 | 8 | 10 | 495 | 6.31 |  | 54 | 8 | 24 | 146 | 4.69 |
|  |  |  | 48 | 10 | -2 |  | 3.88 |  | 54 | 10 | 18 |  | 4.90 |  | 44 | 2 | 22 |  | 3.90 |
|  |  |  |  |  |  |  |  |  | 58 | 2 | -2 |  | 3.60 |  | 50 | 6 | 12 |  | 3.57 |
|  | R Inferior Frontal | 46 |  |  |  |  |  |  | 38 | 36 | 2 | 172 | 4.68 |  | 46 | 38 | 10 | 214 | 4.88 |
|  |  |  |  |  |  |  |  |  | 48 | 28 | 2 |  | 3.97 |  | 42 | 36 | 20 |  | 4.34 |
|  |  |  |  |  |  |  |  |  | 46 | 38 | 16 |  | 3.59 |  |  |  |  |  |  |
|  | L Mid Frontal | 9/10 |  |  |  |  |  |  | -22 | 46 | 32 | 239 | 4.73 |  |  |  |  |  |  |
|  |  |  |  |  |  |  |  |  | -28 | 44 | 24 |  | 4.08 |  |  |  |  |  |  |
|  |  |  |  |  |  |  |  |  | -22 | 54 | 24 |  | 3.69 |  |  |  |  |  |  |
|  | R Mid/Superior Frontal | 9/8 |  |  |  |  |  |  | 34 | 28 | 38 | 196 | 4.68 |  | 18 | 36 | 48 | 194 | 4.56 |
|  |  |  |  |  |  |  |  |  | 22 | 42 | 36 |  | 4.18 |  | 22 | 40 | 34 |  | 3.86 |
|  |  |  |  |  |  |  |  |  | 24 | 24 | 30 |  | 4.12 |  | 24 | 22 | 54 |  | 3.53 |
|  | R Superior Frontal | 6 |  |  |  |  |  |  |  |  |  |  |  |  | 30 | 4 | 60 | 173 | 4.79 |
|  |  |  |  |  |  |  |  |  |  |  |  |  |  |  | 20 | 12 | 58 |  | 4.38 |
|  |  |  |  |  |  |  |  |  |  |  |  |  |  |  | 32 | 14 | 56 |  | 3.68 |
|  | L Supplementary Motor Area (SMA) | 6 | -8 | 2 | 64 | 1162 | 6.41 |  | -4 | 0 | 66 | 1306 | 6.04 |  | -14 | 4 | 62 | 287 | 5.85 |
|  |  |  | 12 | 6 | 62 |  | 5.34 |  | 6 | 6 | 62 |  | 5.97 |  | -4 | 6 | 62 |  | 5.83 |
|  |  |  | 24 | 0 | 56 |  | 5.07 |  | 14 | 0 | 68 |  | 5.36 |  | -8 | -2 | 58 |  | 4.37 |
|  | L Mid Cingulum | 32 |  |  |  |  |  |  | -10 | 8 | 40 | 95 | 4.22 |  |  |  |  |  |  |
|  | L Mid Cingulum |  |  |  |  |  |  |  | -6 | 16 | 38 |  | 4.06 |  |  |  |  |  |  |
| Parietal | L Posterior Cingulum | 23 | -8 | -52 | 28 | 413 | 6.63 |  | -4 | -50 | 28 | 239 | 4.57 |  | -4 | -52 | 26 | 673 | 7.00 |
|  |  |  | 8 | -52 | 26 |  | 5.33 |  | 8 | -52 | 26 |  | 3.94 |  | 8 | -52 | 26 |  | 6.62 |
|  |  |  |  |  |  |  |  |  |  |  |  |  |  |  | 14 | -42 | 20 |  | 3.50 |
|  | R Supramarginal | 40 | 54 | -30 | 44 | 888 | 6.24 |  | 48 | -42 | 54 | 721 | 6.04 |  | 54 | -32 | 48 | 557 | 6.21 |
|  |  |  | 46 | -38 | 42 |  | 6.05 |  | 54 | -36 | 48 |  | 5.92 |  | 48 | -38 | 54 |  | 4.43 |
|  |  |  | 48 | -44 | 54 |  | 4.57 |  | 42 | -40 | 42 |  | 4.87 |  | 44 | -46 | 54 |  | 4.26 |
|  | Precuneus |  |  |  |  |  |  |  | -22 | -44 | 18 | 105 | 4.66 |  |  |  |  |  |  |
|  | L Postcentral | 4 | -38 | -28 | 58 | 290 | 5.33 |  |  |  |  |  |  |  | 2 | -32 | 60 | 229 | 5.49 |
|  |  |  | -40 | -18 | 54 |  | 4.41 |  |  |  |  |  |  |  |  |  |  |  |  |
|  |  |  | -44 | -36 | 54 |  | 3.37 |  |  |  |  |  |  |  |  |  |  |  |  |
|  | R Calcarine | 17/ | 22 | -92 | 0 | 171 | 6.45 |  | 14 | -80 | 4 | 402 | 5.36 |  | 12 | -78 | 4 | 398 | 6.30 |
|  |  | 18 | 16 | -80 | 4 |  | 4.21 |  | 6 | -84 | 2 |  | 4.82 |  | 10 | -72 | -6 |  | 3.45 |
|  |  |  |  |  |  |  |  |  | 6 | -74 | -6 |  | 4.57 |  |  |  |  |  |  |

*Note.* Names were based on the Automatic Anatomical Labeling (AAL) atlas.

**Table S6**

*Regression analyses with Self-concept clarity and Fear of negative evaluation*

|  | **SCC T2** | | | **SCC T3** | | | **FNE T2** | | | **FNE T3** | | |
| --- | --- | --- | --- | --- | --- | --- | --- | --- | --- | --- | --- | --- |
|  | β | *t* | *p* | β | *t* | *p* | β | *t* | *p* | β | *t* | *p* |
| **Positivity T1** |  |  |  |  |  |  |  |  |  |  |  |  |
| Direct self | 0,420 | 2,575 | ***0,011*** | 0,410 | 2,455 | ***0,015*** | -0,206 | -1,235 | 0,219 | -0,351 | -2,057 | ***0,042*** |
| Reflected self | -0,282 | -1,731 | 0,086 | -0,213 | -1,271 | 0,206 | 0,226 | 1,354 | 0,178 | 0,352 | 2,058 | ***0,041*** |
|  |  |  |  |  |  |  |  |  |  |  |  |  |
| Direct academic | 0,360 | 2,149 | ***0,033*** | 0,402 | 2,345 | ***0,020*** | -0,070 | -0,421 | 0,675 | -0,260 | -1,521 | 0,131 |
| Direct physical | 0,350 | 2,438 | ***0,016*** | 0,159 | 1,118 | 0,265 | -0,496 | -3,515 | **0,001*** | -0,503 | -3,502 | **0,001*** |
| Direct prosocial | -0,001 | -0,009 | 0,993 | 0,145 | 1,143 | 0,255 | 0,160 | 1,223 | 0,223 | 0,031 | 0,242 | 0,809 |
| Reflected academic | -0,388 | -2,263 | ***0,025*** | -0,422 | -2,402 | ***0,018*** | 0,180 | 1,058 | 0,292 | 0,464 | 2,640 | ***0,009*** |
| Reflected physical | -0,167 | -1,180 | 0,240 | 0,119 | 0,836 | 0,405 | 0,419 | 3,007 | ***0,003*** | 0,349 | 2,449 | ***0,016*** |
| Reflected prosocial | 0,051 | 0,371 | 0,711 | -0,041 | -0,310 | 0,757 | -0,098 | -0,717 | 0,474 | -0,078 | -0,587 | 0,558 |
|  |  |  |  |  |  |  |  |  |  |  |  |  |
| **mPFC activation T1** |  |  |  |  |  |  |  |  |  |  |  |  |
| Direct > control | -0,111 | -0,882 | 0,379 | -0,046 | -0,354 | 0,724 | 0,099 | 0,793 | 0,429 | 0,058 | 0,444 | 0,658 |
| Reflected > control | 0,027 | 0,219 | 0,827 | 0,115 | 0,885 | 0,378 | -0,072 | -0,576 | 0,566 | -0,143 | -1,096 | 0,275 |
|  |  |  |  |  |  |  |  |  |  |  |  |  |
| Direct academic | -0,049 | -0,284 | 0,777 | 0,014 | 0,079 | 0,937 | -0,021 | -0,119 | 0,906 | 0,002 | 0,009 | 0,992 |
| Direct physical | 0,059 | 0,415 | 0,679 | -0,142 | -0,965 | 0,336 | 0,058 | 0,407 | 0,685 | 0,031 | 0,206 | 0,837 |
| Direct prosocial | -0,144 | -0,762 | 0,448 | 0,117 | 0,601 | 0,549 | 0,087 | 0,459 | 0,647 | 0,041 | 0,208 | 0,836 |
| Reflected academic | -0,104 | -0,640 | 0,523 | -0,198 | -1,198 | 0,233 | -0,095 | -0,582 | 0,562 | 0,082 | 0,493 | 0,623 |
| Reflected physical | -0,018 | -0,112 | 0,911 | 0,132 | 0,796 | 0,427 | -0,009 | -0,056 | 0,956 | 0,038 | 0,230 | 0,819 |
| Reflected prosocial | 0,177 | 1,113 | 0,268 | 0,139 | 0,855 | 0,394 | 0,012 | 0,078 | 0,938 | -0,282 | -1,730 | 0,086 |
|  |  |  |  |  |  |  |  |  |  |  |  |  |

*Note.* All regression analyses were controlled for Age at T1. **Bold*** = survives multiple comparisons correction. ***Bold italic*** = does not survive multiple comparisons correction.

**Table S7**

*Regressions with developmental slopes of Self-concept clarity and Fear of negative evaluation*

|  | **Slope SCC** | | | **Slope FNE** | | |
| --- | --- | --- | --- | --- | --- | --- |
|  | β | *t* | *p* | β | *t* | *p* |
| **Positivity Slope** |  |  |  |  |  |  |
| Direct self | 0,356 | 2,869 | **0,005*** | 0,004 | 0,044 | 0,965 |
| Reflected self | -0,225 | -1,873 | 0,063 | -0,187 | -1,449 | 0,150 |
|  |  |  |  |  |  |  |
| Direct academic | 0,094 | 0,746 | 0,457 | -0,044 | -0,324 | 0,747 |
| Direct physical | 0,461 | 4,157 | **0,000*** | -0,192 | -1,606 | 0,111 |
| Direct prosocial | -0,092 | -0,930 | 0,354 | -0,023 | -0,219 | 0,827 |
| Reflected academic | -0,100 | -0,826 | 0,410 | 0,085 | 0,652 | 0,515 |
| Reflected physical | -0,163 | -1,565 | 0,120 | -0,053 | -0,468 | 0,640 |
| Reflected prosocial | -0,063 | -0,599 | 0,550 | 0,194 | 1,719 | 0,088 |
| **mPFC activation Slope** | | |  |  |  |  |
| Direct > control | 0,025 | 0,191 | 0,849 | -0,024 | -0,180 | 0,857 |
| Reflected > control | -0,082 | -0,642 | 0,522 | 0,073 | 0,557 | 0,579 |
|  |  |  |  |  |  |  |
| Direct academic | 0,035 | 0,201 | 0,841 | -0,067 | -0,380 | 0,705 |
| Direct physical | 0,151 | 1,034 | 0,303 | -0,121 | -0,815 | 0,417 |
| Direct prosocial | -0,189 | -1,064 | 0,289 | 0,162 | 0,895 | 0,373 |
| Reflected academic | 0,184 | 1,204 | 0,231 | -0,119 | -0,766 | 0,445 |
| Reflected physical | -0,133 | -0,826 | 0,410 | -0,058 | -0,357 | 0,722 |
| Reflected prosocial | -0,094 | -0,557 | 0,578 | 0,237 | 1,375 | 0,172 |
|  |  |  |  |  |  |  |

*Note.* All regression analyses were controlled for Age at T1. **Bold*** = survives multiple comparisons correction. ***Bold italic*** = does not survive multiple comparisons correction.
