# Supplementary material for: Longitudinal self-concept development in adolescence
Source: Soc Cogn Affect Neurosci. 2023 Jan 14;18(1):nsac062. doi: 10.1093/scan/nsac062 (PMC10036877; doi:10.1093/scan/nsac062)
Supplement: nsac062_Supp [file nsac062_supp.zip › scan-22-038-File003.docx]

**SI Task stimuli**

Stimuli used in the Direct and Reflected self condition, and in the control condition.

| **Direct self task** | **Reflected self task** | **Control task** |
| --- | --- | --- |
|  | **Anderen denken over mij dat…** |  |
| **Academic Positive** | |  |
| Ik ben gemotiveerd op school | …ik gemotiveerd ben op school | Mooie ogen hebben |
| Ik ben goed in het maken van toetsen | …ik goed ben in het maken van toetsen | Lage cijfers halen |
| Ik leer graag | …ik graag leer | Gemeen zijn tegen anderen |
| Ik leer snel | …ik snel leer | Mollig zijn |
| Ik ben slim | …ik slim ben | Spelfouten maken |
| Ik doe het goed op school | …ik het goed doe op school | Anderen buitensluiten |
| Ik heb mijn werk altijd op tijd klaar | …ik mijn werk altijd op tijd klaar heb | Een gezond gewicht hebben |
| Ik ben een harde werker | …ik een harde werker ben | Netjes werken |
| Ik haal goede cijfers | …ik goede cijfers haal | Anderen pesten |
| Ik werk zelfstandig | …ik zelfstandig werk | Ruzie maken |
| **Academic Negative** | | Anderen vergeven |
| Ik stel dingen uit | …ik dingen uitstel | Spullen delen met anderen |
| Ik heb veel hulp nodig op school | …ik veel hulp nodig heb op school | Er moe uit zien |
| Ik ben lui | …ik lui ben | Langzaam lezen |
| Ik werk sloom | …ik sloom werk | Puistjes hebben |
| Ik ben onverstandig | …ik onverstandig ben | Ruzies oplossen |
| Ik werk chaotisch | …ik chaotisch werk | Tevreden zijn met je uiterlijk |
| Ik vind school moeilijk | …ik school moeilijk vind | Een goed geheugen hebben |
| Ik ben snel afgeleid | …ik snel afgeleid ben | Een doorzetter zijn |
| Ik werk slordig | …ik slordig werk | Goed zijn in rekenen |
| Ik ben dom | …ik dom ben |  |
| **Physical Positive** | |  |
| Ik ben mooi | …ik mooi ben |  |
| Ik zie er stralend uit | …ik er stralend uit zie |  |
| Ik ben knap | …ik knap ben |  |
| Ik heb een goed lichaam | …ik een goed lichaam heb |  |
| Ik heb een mooie lach | …ik een mooie lach heb |  |
| Ik heb een goede kledingstijl | …ik een goede kledingstijl heb |  |
| Ik zie er aantrekkelijk uit | …ik er aantrekkelijk uit zie |  |
| Ik mag blij zijn met mijn lichaam | …ik blij mag zijn met mijn lichaam |  |
| Ik zie er goed uit | …ik er goed uit zie |  |
| Ik heb een mooi figuur | …ik een mooi figuur heb |  |
| **Physical Negative** | |  |
| Ik ben te zwaar | …ik te zwaar ben |  |
| Ik ben lelijk | …ik lelijk ben |  |
| Ik heb een slechte huid | …ik een slechte huid heb |  |
| Ik zweet veel | …ik veel zweet |  |
| Ik ben dik | …ik dik ben |  |
| Ik zie er suf uit | …ik er suf uit zie |  |
| Ik ben onaantrekkelijk | …ik onaantrekkelijk ben |  |
| Ik heb overgewicht | …ik overgewicht heb |  |
| Ik zie er onverzorgd uit | …ik er onverzorgd uit zie |  |
| Ik heb lelijke tanden | …ik lelijke tanden heb |  |
| **Prosocial Positive** | |  |
| Ik leef met anderen mee | …ik met anderen meeleef |  |
| Ik troost anderen | …ik anderen troost |  |
| Ik houd rekening met anderen | …ik rekening houd met anderen |  |
| Ik help anderen | …ik anderen help |  |
| Ik voel met anderen mee | …ik met anderen meevoel |  |
| Ik geef om anderen | …ik om anderen geef |  |
| Ik kom voor anderen op | …ik voor anderen opkom |  |
| Ik zorg graag voor anderen | …ik graag voor anderen zorg |  |
| Ik doe graag iets voor een ander | …ik graag iets voor een ander doe |  |
| Ik deel graag met anderen | …ik graag met anderen deel |  |
| **Prosocial Negative** | |  |
| Ik kies altijd voor mezelf | …ik altijd voor mezelf kies |  |
| Ik laat anderen hun problemen zelf oplossen | …ik anderen hun problemen zelf laat oplossen |  |
| Ik leen mijn spullen niet graag uit | …ik mijn spullen niet graag uitleen |  |
| Ik houd alleen rekening met mezelf | …ik alleen rekening houd met mezelf |  |
| Ik denk vooral aan mezelf | …ik vooral aan mezelf denk |  |
| Ik help anderen alleen als ik er iets voor terug krijg | …ik alleen anderen help als ik er iets voor terug krijg |  |
| Ik zorg alleen voor mezelf | …ik alleen voor mezelf zorg |  |
| Ik negeer andermans problemen | …ik andermans problemen negeer |  |
| Ik help nooit een vreemde | …ik nooit een vreemde help |  |
| Ik houd alles voor mezelf | …ik alles voor mezelf houd |  |

**SI Task stimuli - English translation**

Stimuli used in the Direct and Reflected self condition, and in the control condition.

| **Direct self task** | **Reflected self task** | **Control task** |
| --- | --- | --- |
|  | Others think about me that... |  |
| Academic Positive | |  |
| I am motivated in school | …I am motivated in school | Having beautiful eyes |
| I am good at taking tests | …I am good at taking tests | Getting low grades |
| I like to learn | …I like to learn | Being mean to others |
| I learn fast | …I learn fast | Being chubby |
| I am smart | …I am smart | Making spelling errors |
| I do well in school | …I do well in school | Exclude others |
| I always finish my work in time | …I always finish my work in time | Having a healthy weight |
| I’m a hard worker | …I’m a hard worker | Work neatly |
| I get good grades | …I get good grades | Bully others |
| I work independently | …I work independently | Arguing |
| Academic Negative | | Forgiving others |
| I procrastinate | …I procrastinate | Sharing stuff with others |
| I need a lot of help at school | …I need a lot of help at school | Looking tired |
| I’m lazy | …I’m lazy | Reading slowly |
| I work slow | …I work slow | Having pimples |
| I am irresponsible | …I am irresponsible | Resolving quarrels |
| I work chaotically | …I work chaotically | Being happy with one’s appearance |
| I think school is difficult | …I think school is difficult | Having a good memory |
| I’m easily distracted | …I’m easily distracted | Having perseverance |
| I’m a messy worker | …I’m a messy worker | Being good at math |
| I’m dumb | …I’m dumb |  |
| Physical Positive | |  |
| I am beautiful | …I am beautiful |  |
| I look radiant | …I look radiant |  |
| I’m pretty (female/other) handsome (male) | …I’m pretty (female/other) handsome (male) |  |
| I have a good body | …I have a good body |  |
| I have a beautiful smile | …I have a beautiful smile |  |
| I am stylish. | …I am stylish. |  |
| I look attractive | …I look attractive |  |
| I should be happy with my body | …I should be happy with my body |  |
| I look good | …I look good |  |
| I have a beautiful figure | …I have a beautiful figure |  |
| Physical Negative | |  |
| I weigh too much | …I weigh too much |  |
| I’m ugly | …I’m ugly |  |
| I have bad skin | …I have bad skin |  |
| I sweat a lot | …I sweat a lot |  |
| I’m fat | …I’m fat |  |
| I look dull | …I look dull |  |
| I’m unattractive | …I’m unattractive |  |
| I’m overweight | …I’m overweight |  |
| I look scruffy | …I look scruffy |  |
| I have ugly teeth | …I have ugly teeth |  |
| Prosocial Positive | |  |
| I empathize with others | …I empathize with others |  |
| I comfort others | …I comfort others |  |
| I am considerate of others | …I am considerate of others |  |
| I help others | …I help others |  |
| I sympathize with others | …I sympathize with others |  |
| I care about others | …I care about others |  |
| I stand up for others | …I stand up for others |  |
| I like to take care of others | …I like to take care of others |  |
| I like doing something for someone else | …I like doing something for someone else |  |
| I like to share with others | …I like to share with others |  |
| Prosocial Negative | |  |
| I am selfish. | …I am selfish. |  |
| I let others solve their problems themselves | …I let others solve their problems themselves |  |
| I don’t like to lend my belongings. | …I don’t like to lend my belongings. |  |
| I am inconsiderate. | …I am inconsiderate. |  |
| I mainly think about myself | …I mainly think about myself |  |
| I only help others when I get something in return | …I only help others when I get something in return |  |
| I only take care of myself | …I only take care of myself |  |
| I ignore other people’s problems | …I ignore other people’s problems |  |
| I never help a stranger | …I never help a stranger |  |
| I keep everything to myself | …I keep everything to myself |  |
