# Supplementary material for: Longitudinal self-concept development in adolescence
Source: Soc Cogn Affect Neurosci. 2023 Jan 14;18(1):nsac062. doi: 10.1093/scan/nsac062 (PMC10036877; doi:10.1093/scan/nsac062)
Supplement: nsac062_Supp [file nsac062_supp.zip › scan-22-038-File004.docx]

**SI Deviations from the preregistration**

The current study was preregistered in a large preregistration including information on the larger Leiden Self-concept study. For that reason, not everything that has been mentioned in the preregistration was included in the current study. Here, we detail deviations between the preregistration and this manuscript.

- The current study was preregistered in topic 1, aim 1. Aim 2 is intended to be a separate manuscript.
- Only the questionnaires mentioned under topic 1, aim 1 are included in the current study. The full list of questionnaires that were filled out in the larger Leiden Self-concept study are mentioned in the preregistration but were not part of the current study.
- We did not include pubertal development in our final analyses, because of its high correlation to age, which was our main predictor of interest. Additionally, there were many missing values on the pubertal development measure, which would prevent us from using all the relevant data that we have available. Future studies should examine this question in samples that are selected based on variation in puberty with age relatively constant (3).
- The first hypothesis did not test for domain differentiation in self-positivity but for general age-trends in self-positivity. The domain differentiation would require a different statistical approach for which the current design was not optimized.
- IQ was not used as a covariate in our analyses because of its correlation with academic self-concept.
- Although not preregistered, we explored random linear age effects to show possible differences in developmental trajectories between participants
- Since our lab recently made the transition to spm12, fMRI data was analyzed using spm12 instead of spm8.
